# Ring-Fused *meso*-Tetraarylchlorins as Auspicious PDT Sensitizers: Synthesis, Structural Characterization, Photophysics, and Biological Evaluation

**DOI:** 10.3389/fchem.2022.873245

**Published:** 2022-04-27

**Authors:** Mafalda Laranjo, Nelson A. M. Pereira, Andreia S. R. Oliveira, Márcia Campos Aguiar, Gonçalo Brites, Bruno F. O. Nascimento, Beatriz Serambeque, Bruna D. P. Costa, João Pina, J. Sérgio Seixas de Melo, Marta Pineiro, M. Filomena Botelho, Teresa M. V. D. Pinho e Melo

**Affiliations:** ^1^ Institute of Biophysics and Institute for Clinical and Biomedical Research (iCBR), Area of Environment Genetics and Oncobiology (CIMAGO), Faculty of Medicine, University of Coimbra, Coimbra, Portugal; ^2^ Centre of Innovative Biomedicine and Biotechnology (CIBB), University of Coimbra, Coimbra, Portugal; ^3^ Clinical and Academic Centre of Coimbra (CACC), Coimbra, Portugal; ^4^ Department of Chemistry, Coimbra Chemistry Centre-Institute of Molecular Sciences, University of Coimbra, Coimbra, Portugal

**Keywords:** chlorins, photodynamic therapy, photosensitizer, subcellular accumulation, cancer

## Abstract

Novel 4,5,6,7-tetrahydropyrazolo[1,5-*a*]pyridine-fused *meso*-tetraarylchlorins, with different degrees of hydrophilicity (with methyl ester, hydroxymethyl, and carboxylic acid moieties), have been synthesized and their photophysical characterization as well as *in vitro* photocytotoxicity assessment against human melanoma and esophageal and bladder carcinomas was carried out. An integrated analysis of the photosensitizers’ performance, considering the singlet oxygen generation data, cell internalization, and intracellular localization, allowed to establish relevant structure-photoactivity relationships and the rationalization of the observed photocytotoxicity. In the diacid and monoalcohol series, chlorins derived from *meso*-tetraphenylporphyrin proved to be the most efficient photodynamic therapy agents, showing IC_50_ values of 68 and 344 nM against A375 cells, respectively. These compounds were less active against OE19 and HT1376 cells, the diacid chlorin with IC_50_ values still in the nano-molar range, whereas the monohydroxymethyl-chlorin showed significantly higher IC_50_ values. The lead di(hydroxymethyl)-substituted *meso*-tetraphenylchlorin confirmed its remarkable photoactivity with IC_50_ values below 75 nM against the studied cancer cell lines. Subcellular accumulation of this chlorin in the mitochondria, endoplasmic reticulum, and plasma membrane was demonstrated.

## 1 Introduction

Photodynamic therapy (PDT) depends on the combined action of oxygen, light, and a suitable photosensitizing chromophore in order to harm or destroy abnormal tissues in cancer and to treat various non-malignant diseases, including pathogenic infections (Dolmans et al., 2003; Abrahamse and Hamblin, 2016; Hamblin, 2016). The photosensitizer (PS) and light are not toxic *per se*, but their combination enables the generation of reactive oxygen species (ROS) from molecular oxygen through photo-induced energy and/or electron transfer processes (Plaetzer et al., 2009). These high-energy ROS, such as singlet oxygen, superoxide radical anion, and hydroxyl radicals, are responsible for promoting damage to relevant cellular machinery, causing powerful cytotoxic effects, impairing related tissue vasculature, and activating an inflammatory reaction and subsequent immune response (Celli et al., 2010). On the specific subject of oncological diseases, since these processes occur in the immediate surroundings of the light-absorbing molecule, exceptional spatial control of cytotoxicity can be achieved *via* targeted and selective accumulation of the PS in tumor tissues (O’Connor et al., 2009; van Straten et al., 2017). This leads to the decrease of the off-target injury resulting in scarcer side-effects, in comparison with the classic and systemic chemotherapy and radiotherapy treatments (Hopper, 2000; Brown et al., 2004). Hence, it comes as no surprise that PDT has accomplished enormous success in treating numerous dermal (Kalka et al., 2000) and ocular (Bressler and Bressler, 2000) conditions, while also being widely considered an appropriate therapeutic strategy in the management of cancers, either by itself or in combination modalities, along with radio-, chemo-, and immunotherapies (Gollnick and Brackett, 2010; Dąbrowski and Arnaut, 2015; Luo et al., 2017).

Despite the various advantages of photonic therapies and diagnostics, the limited penetration of visible light into living tissues is critical. Nevertheless, this drawback can be bypassed by using adequate excitation light and PS that strongly absorb between 600 and 1,300 nm, i.e., above the absorption of heme and below the absorption of water, respectively. While near infrared (NIR) wavelengths longer than 850 nm do not deliver sufficient energy for the efficient production of ROS, light within the phototherapeutic window (600–850 nm) is able to efficiently produce ROS and reach several centimeters of tissue (Frangioni, 2003; Ntziachristos et al., 2003; Juzeniene et al., 2007; Wilson and Patterson, 2008). Additionally, the use of NIR light lessens tissue autofluorescence, the inherent fluorescence of tissues owing to endogenous fluorophores, such as elastin, collagen, and flavins (Ntziachristos et al., 2003; Juzeniene et al., 2007). An ideal PS must also exhibit a considerable triplet quantum yield, which enables a good production of ROS upon irradiation. It should present very low or no toxicity in the dark and fairly fast clearance from healthy tissues, thus curtailing the well-known side-effects of phototoxicity (Ferrand et al., 2003).

Chlorins (dihydroporphyrins) are renowned members of the tetrapyrrolic macrocyclic family, and are commonly characterized by a strong and red-shifted absorbance within the phototherapeutic window and good ROS production yields, thus being widely recognized as a better PSs for PDT than porphyrins (Bonnett et al., 1989; Gomer, 1991; Ris et al., 1991; Berenbaum et al., 1993; Ferrand et al., 2003; Banfi et al., 2004; Dąbrowski et al., 2010; Senge and Brandt, 2011; Tanaka et al., 2014). In fact, chlorins are currently well represented in the rather circumscribed group of clinically approved PSs, e.g., *meso*-tetra(*m*-hydroxyphenyl)chlorin (mTHPC, temoporfin, Foscan®), approved in the European Union (EU), Norway, and Iceland since 2001 for advanced head and neck cancer; benzoporphyrin derivative monoacid ring A (BPD-MA, verteporfin, Visudyne®), approved in over 70 countries since 2001 for age-related macular degeneration; and mono-L-aspartyl chlorin e6 (NPe6, talaporfin, Laserphyrin®), approved in Japan since 2004 for early and centrally located lung cancer (Liu et al., 2014; van Straten et al., 2017). Chlorins are easily derived from porphyrin precursors by simple reduction, but exhibit restricted stability issues due to the ease of back oxidation to the porphyrin state and photobleaching (Senge and Brandt, 2011; Senge, 2012). One approach to minimize these problems is the preparation of geminal dialkylated β-substituted chlorins, because the unwanted dehydrogenation process is blocked, although this entails the complex synthetic methodologies (Balasubramanian et al., 2000; Strachan et al., 2000; Kim and Lindsey, 2005). An easier strategy to synthesize chlorins is through cycloaddition reactions on the porphyrin nucleus, namely, 1,3-dipolar and Diels–Alder cycloadditions (Fox and Boyle, 2006; Galezowski and Gryko, 2007; Pineiro et al., 2012). “Locked” chlorins with intensified stability can be achieved by means of 1,3-dipolar cycloaddition of the porphyrins bearing two vicinal electron-withdrawing groups at the β positions (Gałȩzowski and Gryko, 2006). Yet, these derivatizations usually yield tetrahydroporphyrins (bacteriochlorins and/or isobacteriochlorins) as unwanted side-products, which complicates the isolation and purification procedures of the target chlorin. As we have previously demonstrated, the stability of chlorins can be greatly enhanced with the simple introduction of a fused ring *via* [8π + 2π] cycloaddition reactions (Pereira et al., 2010; Pereira et al., 2011). In fact, it was shown that porphyrins react with *in situ* generated diazafulvenium methides to deliver a new type of stable 4,5,6,7-tetrahydropyrazolo[1,5-*a*]pyridine-fused chlorins with favorable photophysical features regarding PDT. Some derivatives have proven to be highly active PSs in different cancer cell lines (Pereira et al., 2015; Pereira et al., 2018; Nascimento et al., 2019; Pereira et al., 2021). A similar synthetic approach has also been recently followed by us in order to obtain novel platinum (II) complexes of these types of ring-fused chlorins, showing an improved NIR luminescence, ratiometric molecular oxygen sensing, and photodynamic action properties *in vitro* and *in vivo*, which make them promising leads for cancer theranostic applications (Pereira et al., 2017; Laranjo et al., 2020).

**SCHEME 1:**
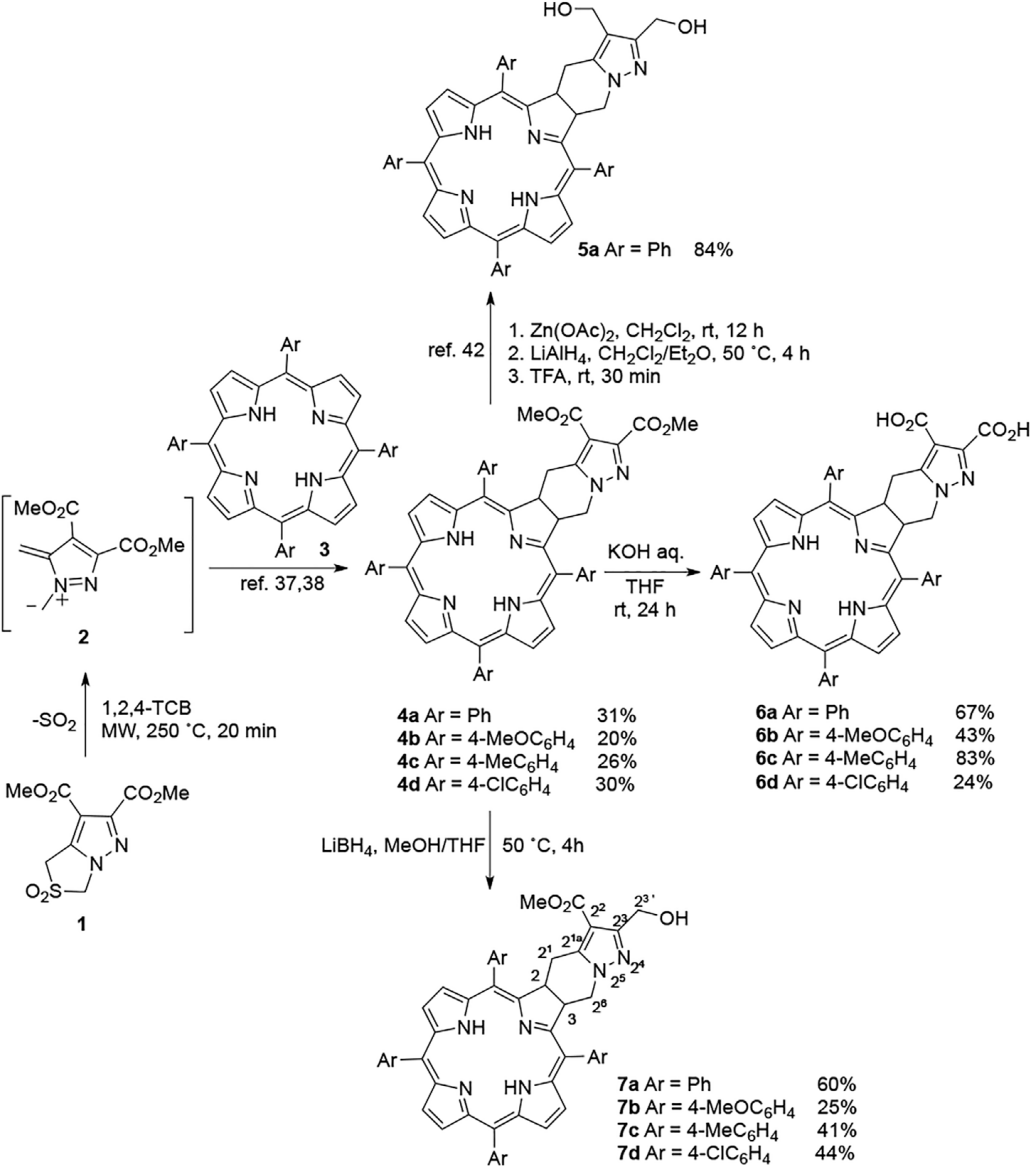
Synthesis of chlorins **4**–**7**.

We observed that increasing the hydrophilicity of these macrocycles is crucial to ensure nanomolar activity against these cells. For instance, dihydroxymethyl chlorin **5a** (Scheme 1) exhibited higher levels of intracellular accumulation and phototoxic activity towards A375 melanoma cells than its corresponding diester-substituted derivative **4a** (Pereira et al., 2015). Notwithstanding the proven and formidable photodynamic performance of some of the studied PSs, it was decided to lengthen the scope of our study by exploring chlorins bearing substituents of different chemical nature, namely hydroxymethyl and carboxylic acid moieties at the exocyclic ring. This was undertaken considering that these structural modulations should lead to PSs with improved proprieties concerning diffusion, distribution, and accumulation in the target tumor tissues. Specifics on the design, synthesis, structural and photophysical characterization, as well as complete *in vitro* biological assessment of a series of new 4,5,6,7-tetrahydropyrazolo[1,5-*a*]pyridine-fused chlorins as PDT agents in human skin malignant melanoma, esophageal adenocarcinoma, and urinary bladder carcinoma cell lines are herein disclosed.

## 2 Results and Discussion

### 2.1 Synthesis

Ring-fused chlorins **4** were obtained *via* [8π + 2π] cycloaddition of diazafulvenium methide **2**, formed *in situ* from sulfur dioxide extrusion of sulfone **1** under microwave irradiation, with porphyrins **3** according to the procedure previously developed by our group (Pereira et al., 2010; Pereira et al., 2011). In order to further explore structure-activity relationships, we decided to expand the study to other derivatives having different amphiphilic properties. Thus, structural modulation of chlorins **4** was carried out to obtain photosensitizers **6** and **7**. In the previous work, we employed LiAlH_4_ to reduce the esters of the ring-fused chlorins to the dihydroxymethyl derivatives (Pereira et al., 2015). In the present article, one of our goals was the synthesis of PSs with intermediate hydrophilic features from those compounds. Therefore, we have selected a weaker reducing agent to afford the monoester reduction of **4**. Based on a mild method described by Soai *et al.* applied in the reduction of esters (Soai and Ookawa, 1986), chlorin **4** was reacted with an excess of lithium borohydride (6 equiv.) in the presence of methanol, at 50°C for 4 h, leading to monohydroxymethyl derivatives **7** in a site selective reduction. Somewhat unexpectedly, only one of the two possible isomers was obtained in all the chlorins bearing different *meso*-aryl groups. This selective reduction has a great potential and could be further explored for the synthesis of PS conjugates for targeted PDT. Following this synthetic approach chlorins **7** were obtained in moderate to good yields (25–60%). The structural elucidation of chlorins **7** was established by ^1^H NMR and ^13^C NMR data supported by heteronuclear two-dimensional HMBC spectra (400 MHz). The HMBC spectrum of compound **7a** (Supplementary Figure S2) showed a more intense correlation between the protons of the hydroxymethyl group (H-2^3′^, Scheme 1) at 4.58 ppm and the carbon at position C-2^3^ (*δ* = 153.7 ppm) than with the carbon C-2^2^ at 107.6 ppm.

Among the most important clinical photosensitizers for PDT, some are based on naturally occurring porphyrinic macrocycles bearing carboxylic acid moieties or their salt derivatives, such as benzoporphyrin derivative monoacid ring A, chlorin e6, mono-L-aspartyl chlorin e6, pyrophaeophorbide derivative (HPPH), Tookad®, ALA-induced protoporphyrin IX, and Photofrin® (Abrahamse and Hamblin, 2016). Additionally, some authors reported the strong evidence that the extracellular–intracellular pH gradient in tumor cells might play an important role in the accumulation and selectivity of sensitizers that possess carboxylic acid chains (Bonneau et al., 2004; Das et al., 2005; Mojzisova et al., 2007). Also, recently we have demonstrated that the carboxylic acid derivatives of Pt(II) ring-fused chlorins were very active against different cancer cell lines (Laranjo et al., 2020). In this context, we have decided to derivatize chlorins **4** to the corresponding diacid derivatives **6** under similar conditions. The hydrolysis reaction was performed with saturated aqueous KOH at room temperature for 24 h, chlorins **6** being obtained in moderate to very good yields (24–83%).

### 2.2 Photophysics

**FIGURE 1:**
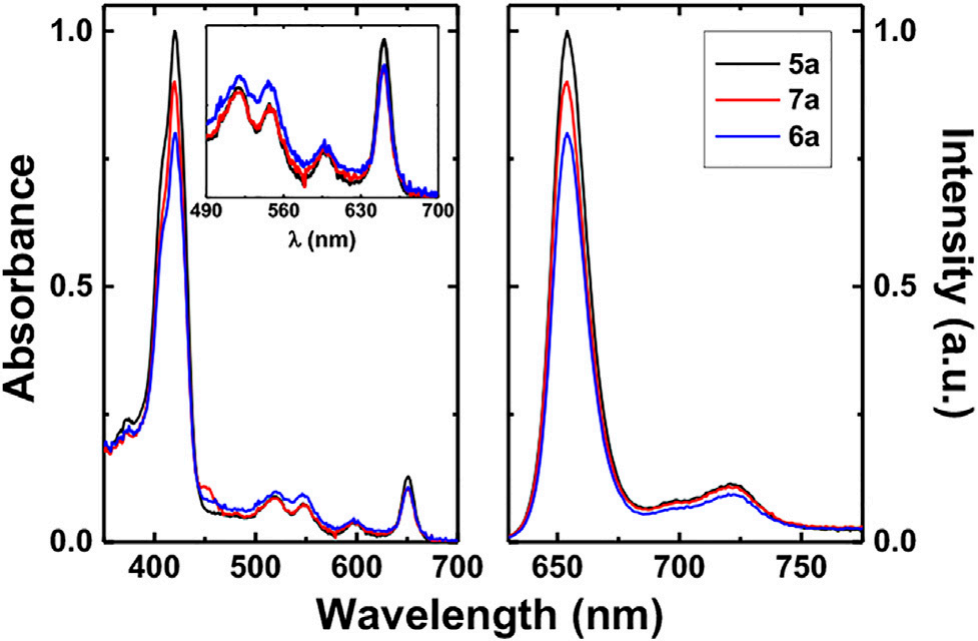
Normalize room temperature absorption (left) and fluorescence emission (right) spectra of chlorins **4a**, **5a**, **6a**, and **7a** in DMSO solution (λ_exc_ = 425 nm).

The absorption and fluorescence emission spectra of representative ring-fused chlorin photosensitizers **5a**, **6a,** and **7a** in dimethylsulfoxide (DMSO) solution are presented in Figure 1. The classical absorption features of the chlorin macrocycle were observed for all new derivatives, with an intense Soret band centered at 420 nm and four Q bands in the visible region, and a more intense band at ∼650 nm standing out. These spectral data are shown in Table 1. In general, similarly structured emission bands were found for the investigated chlorins with wavelength maxima at ca. 654 and 720 nm. Fluorescence quantum yields (ϕ_F_) were obtained by the comparative method using *meso*-tetraphenylporphyrin (TPP) as the reference compound (Montalti et al., 2006). Within the experimental error, the ϕ_F_ values were found to remain constant for the studied ring-fused chlorins (ϕ_F_ ∼0.30 for **4a**, ϕ_F_ ∼0.36 for **5a**, ∼0.34 for **6a**, and ∼0.33 for **7a**), hence showing that the existence of methyl ester, hydroxymethyl, and carboxylic acid moieties at the exocyclic ring does not affect the emission properties.

**TABLE 1:** Absorption coefficients (ε), fluorescence quantum yields (ϕF) and singlet oxygen formation quantum yields (ϕΔ) of chlorins **4a**, **5a**, **6** and **7** in DMSO.

Chlorin	Absorption λ (nm), ε(M^-1^ cm^-1)^					ΦF	^Φ^Δ
	B(0-0)	Qy(1-0)	Qy(0-0)	Qx(1-0)	Qx(0-0)		
**4a**	420, 5.4x10^4^	520, 5.0x10^3^	546, 4.3x10^3^	597, 2.1x10^3^	649, 8.2x10^3^	0.30	0.64
**5a**	**420, 5.1x10^4^**	**518, 4.4x10^3^**	**547, 3.2x10^3^**	**597, 1.8x10^3^**	**650, 5.6x10^3^**	**0.36**	**0.34**
**6a**	**421, 5.8x10^4^**	**521, 3.8x10^3^**	**548, 3.2x10^3^**	**597, 1.6x10^3^**	**650, 6.1x10^3^**	**0.34**	**0.47**
**6b**	425, 5.2x10^4^	525, 4.7x10^3^	553, 4.9x10^3^	598, 2.3x10^3^	651, 7.0x10^3^	0.35	0.33
**6c**	425, 5.7x10^4^	525, 4.2x10^3^	553, 3.8x10^3^	598, 1.9x10^3^	651, 6.6x1^3^	0.36	0.33
**6d**	425, 5.1x10^4^	525, 3.8x10^3^	553, 2.6x10^3^	598, 1.5x10^3^	651, 4.4x10^3^	0.28	0.32
**7a**	**420, 5.5x10^4^**	**520, 5.2x10^3^**	**546, 4.4x10^3^**	**597, 2.2x10^3^**	**649, 8.4x10^3^**	**0.33**	**0.52**
**7b**	425, 5.4x10^4^	523, 4.5x10^3^	554, 4.6x10^3^	597, 2.2x10^3^	650, 6.1x10^3^	0.41	0.37
**7c**	425, 4.9x10^4^	523, 4.2x10^3^	554, 3.6x10^3^	597, 1.8x10^3^	650, 5.6x10^3^	0.41	0.30
**7d**	421, 5.4x10^4^	519, 4.7x10^3^	548, 3.7x10^3^	597, 1.8x10^3^	651, 7.5x10^3^	0.33	0.35

In order to assess the potential of these new free-base ring-fused *meso*-tetraphenylchlorin derivatives as PDT agents, singlet oxygen (^1^O_2_) sensitization quantum yields (ϕ_Δ_) were attained by direct measurement of the distinctive phosphorescence emission of ^1^O_2_, following photosensitization of aerated DMSO solutions of the molecules. This was done *via* a comparative method, using TPP in toluene as a reference PS, by plotting the initial phosphorescence intensity (at 1,270 nm) as a function of the laser dose, and comparing the slope with that obtained for the reference compound, the ϕ_Δ_ values of all the PSs are collected in Table 1. ϕ_Δ_ values of 0.64 (**4a**), 0.34 (**5a**), 0.47 (**6a**), and 0.52 (**7a**) were thus found, suggesting that the presence of carboxylic acid and methyl ester functionalities in the exocyclic ring substituted chlorins significantly increases the ^1^O_2_ photosensitization process. This pattern was previously observed for 4,5,6,7-tetrahydropyrazolo[1,5-*a*]pyridine-fused 5,15-diphenylchlorins where a significant decrease was observed in the singlet oxygen formation quantum yields going from the ester derivatives (values in the 0.66–0.70 range) to the corresponding dihydroxymethyl derivatives (values lying between 0.19 and 0.27) (Pereira et al., 2018). The introduction of methoxy, methyl, or chlorine substituents (**6b**–**d** and **7b**–**d**) at the *para* position of the phenyl rings does not significantly influence the photophysical parameters of these compounds.

### 2.3 Photocytotoxicity

**FIGURE 2:**
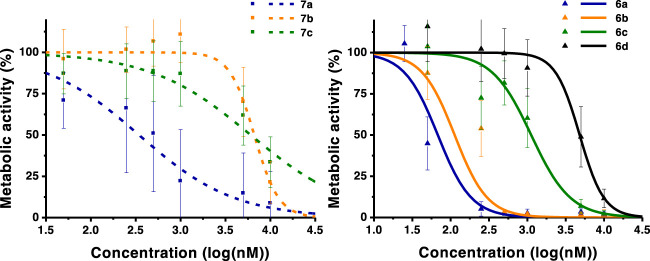
Dose-response curves of A375 skin malignant melanoma cells. Analysis performed 24 h after PDT. The graph in the left includes monohydroxymethyl derivatives **7a**–**c**. The graph in the right includes diacid chlorins **6**. Data points represent the mean ±SD.

**FIGURE 3:**
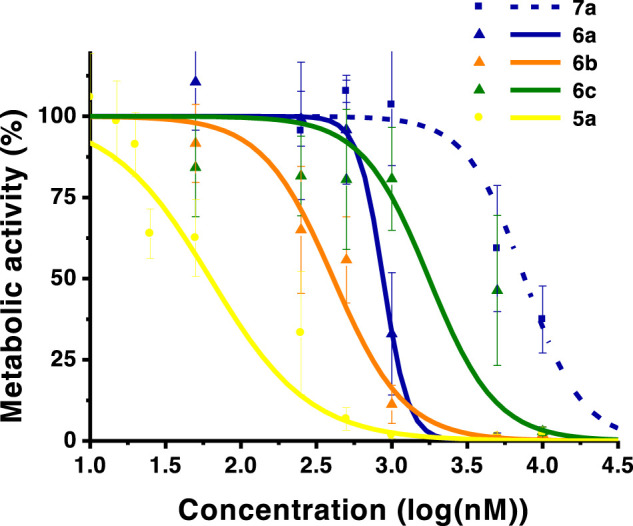
Dose-response curves of OE19 esophageal adenocarcinoma cells. Analysis performed 24 h after PDT. The graph includes monohydroxymethyl derivative **7a**, diacid chlorins **6a–c**, and the dihydroxymethyl compound **5a**. Data points represent the mean ± SD.

**FIGURE 4:**
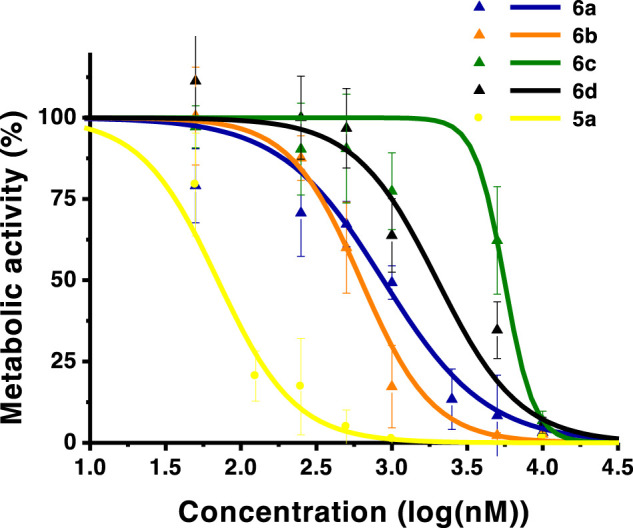
Dose-response curves of HT1376 urinary bladder carcinoma cells. Analysis performed 24 h after PDT. The graph includes diacid chlorins **6a**, **6b**, **6c**, and **6d**, and dihydroxymethyl compound **5a**. Data points represent the mean ±SD.

The photocytotoxicity of the new ring-fused *meso*-tetraarylchlorins was evaluated against human skin malignant melanoma (A375), esophageal adenocarcinoma (OE19), and urinary bladder carcinoma (HT1376) cell lines (Table 2; Figures 2, 3, 4). An IC_50_ value of 31 nM against A375 cells was previously determined for the lead di(hydroxymethyl)-substituted *meso*-tetraphenylchlorin **5a** (Pereira et al., 2015). The high efficacy of this compound as a PDT therapeutic agent was corroborated by the observed IC_50_ values of 63 and 73 nM against OE19 and HT1376, respectively (Table 2). When comparing with the diester derivative **4a**, it is possible to attribute the huge increase of activity observed for chlorin **5a** to the presence of two hydroxymethyl groups at the 4,5,6,7-tetrahydropyrazolo[1,5-*a*]pyridine-ring system. To assess the influence of other exocyclic ring system substitution patterns on the capabilities of these chlorins to act as photosensitizers, diacid and monoalcohol derivatives were evaluated. The diacid **6a** and monoalcohol derivative **7a** proved to be very active against A375 cells, showing IC_50_ values of 68 and 344 nM, respectively. However, these compounds were less active against OE19 and HT1376 cells, with chlorin **6a** showing IC_50_ values in the 875–878 nM range and chlorin **7a** with significant higher IC_50_ values. Replacing the hydrogen atoms at the *para* positions of the phenyl rings by methoxy (**6** and **7b**), methyl (**6c** and **7c**), or chlorine (**6d** and **7d**) substituents leads to the progressive decrease in the capacity to act as photosensitizers against A375 cells. Hence, the lead dihydroxymethyl derivative **5a** remains the most promising PDT agent within these free-base ring-fused chlorin series. The cytotoxicity in the absence of light was evaluated for derivative **6b**, in all cell lines studied, and no toxicity was observed in concentrations up to 10 μM (Figure S10).

**TABLE 2:** IC_50_ values and confidence intervals at 95% (CI_95_) values of chlorins **4a**, **5a**, **6,** and **7** in human A375 skin malignant melanoma, OE19 esophageal adenocarcinoma, and HT1376 urinary bladder carcinoma cells. Analysis performed 24 h after PDT with red light (cut off < 560 nm) and energy of 10 J. Values, presented in nM, were determined by dose-response sigmoidal fitting (*r* ^2^ > 0.85).

Photocytotoxicity (nM)						
	A375		OE19		HT1376	
Chlorin	IC_5O_	CI_95_	IC_50_	CI_95_	IC_50_	CI_95_
**4a**	1850^a^	(1,090; 3,130)^a^	>10,000		>10,000	
**5a**	31^a^	(24; 41)^a^	63	(37; 106)	73	(34; 152)
**6a**	68	(28; 168)	875	(653; 1,173)	878	(596; 1,293)
**6b**	113	(23; 552)	409	(194; 865)	624	(404; 965)
**6c**	1,122	(750; 1,677)	1762	(504; 6,166)	5,576	(3,743; 8,305)
**6d**	4,806	(3,116; 7,414)	>10,000		1998	(778; 5,131)
**7a**	344	(124; 952)	7,591	(3,632; 15,865)	>10,000	
**7b**	6,612	(5,176; 8,447)	>10,000		>10,000	
**7c**	6,036	(1922; 18,951)	>10,000		>10,000	
**7d**	>10,000		>10,000		>10,000	

a Data previously published in reference 42. CI_95_ meaning confidence intervals at 95%.

**FIGURE 5:**
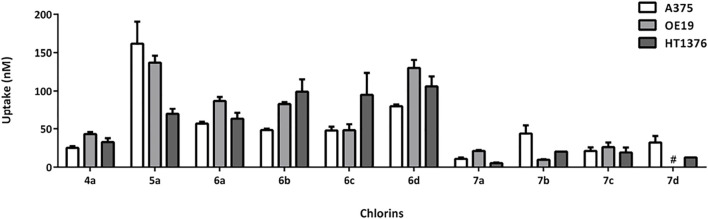
Uptake values of chlorins **4a**, **5a**, **6**, and **7** by human A375 skin malignant melanoma, OE19 esophageal adenocarcinoma, and HT1376 urinary bladder carcinoma cells. Cells were incubated with the chlorins in a concentration of 500 nM for 24 h. Results are presented as mean ±SE.

The decrease of photocytotoxicity in the following order **5a** > **6a** > **7a** > **4a** does not correspond to the capability to produce singlet oxygen, since **5a** is the PS with the lowest ϕ_Δ_ value. Therefore, the cellular uptake of the photosensitizers was evaluated (Figure 5). Two different groups are clearly distinctive, where the influence of the substitution at the exocyclic ring is evident: the PSs with one or two methyl ester substituents (**4a** and **7**), which present cellular uptake values below 50 nM, and PSs with two hydroxymethyl or carboxylic acid substituents (**5a** and **6**), which present cellular uptake values above 50 nM. The diester-substituted derivative **4a** was found to be internalized by the cell cultures in a concentration below 50 nM when initially exposed to 500 nM. Identical results were observed for monohydroxymethyl chlorins **7**, allowing to rationalize the lower photocytotoxic activity of these chlorin derivatives. Therefore, the slight increase in the chlorins’ hydrophilicity prompted by the mono-reduction, going from chlorin **4a** to chlorin **7a**, was not sufficient to improve cell uptake. Regarding diacid chlorins **6**, it was observed that these compounds present intermediate cell uptake values (50 nM < uptake <120 nM). Furthermore, it was confirmed that the conversion of the two methyl ester groups into hydroxymethyl functionalities (**5a**) leads to an improvement in cell uptake, not only in A375 skin malignant melanoma cells (Pereira et al., 2015) but also in OE19 and HT1376 cells with values of 137 and 69 nM, respectively.

Therefore, the cellular uptake order **5a** > **6a** > **7a** correlates with the observed photocytotoxicity, making clear the influence of the substitution at the exocyclic ring in the internalization and activity. Despite the less bulky structure of *meso*-tetraphenylchlorin **6a**, the highest cellular uptake within these carboxylic acid-bearing PSs was observed for *meso*-(4-chlorophenyl) derivative **6d**. Thus, the substitution at the phenyl ring influences the uptake but the higher concentration of PS **6d** inside the cell is not enough to balance the low singlet oxygen generation values, resulting in moderate photodynamic activity. Overall, A375 melanoma cells were clearly the most susceptible to PDT, when compared to the other cell lines studied in this work, a fact that is supported by the highest photocytotoxicity data obtained associated with good internalization values in these cells.

**FIGURE 6:**
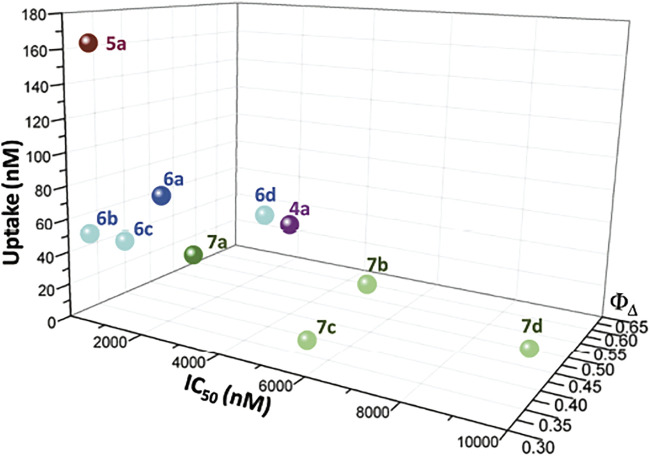
Plot of the singlet oxygen formation quantum yields, cell uptake, and IC_50_ values in human A375 skin malignant melanoma cells of chlorins **4a**, **5a**, **6**, and **7**.

Plotting together the singlet oxygen generation data, cell uptake, and IC_50_ values, Figure 6, the influence of the diacid substitution on the increase of the uptake is evident, whereas the substitution at the phenyl ring has a less pronounced effect on the uptake and does not influence it in a similar way in both series (diacid and monoalcohol). The substitution at the phenyl ring has a negative influence on the capability to produce singlet oxygen. However, in both series there is a correlation between this substitution and the photocytotoxicity, the order a > b > c > d being observed. This effect is not attributable to any of the parameters separately, but to their combination.

### 2.4 Intracellular Distribution

**FIGURE 7:**
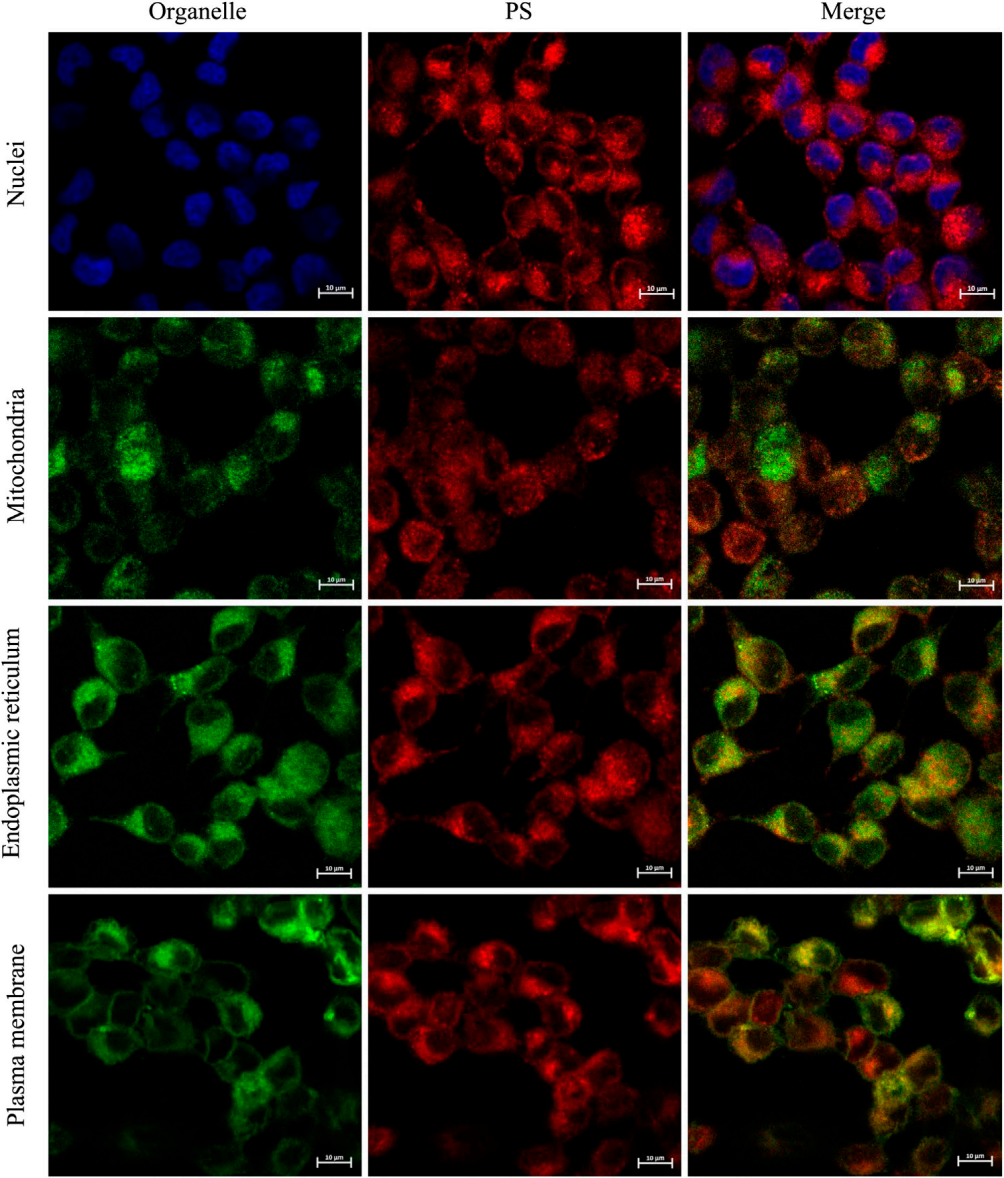
Representative images of chlorin **5a** colocalization in the nuclei, mitochondria, endoplasmic reticulum, and plasma membrane of human A375 skin malignant melanoma cells (for each set of images, subcellular localization of the appropriated probe, of chlorin **5a** and the corresponding merge images are shown). Cells were incubated with chlorin **5a** in a concentration of 500 nM during 24 h.

Chlorin **5a** colocalization in several organelles of A375 melanoma cells is shown in Figure 7. Chlorin **5a** seems unable to surpass the nuclear envelope, as all images analyzed showed a negative Pearson correlation with the nuclear labeling. Nevertheless, distributions in the cytoplasm seem ubiquitous, as strong correlation was found in the membranous organelles. Colocalization with chlorin **5a** showed 78 ± 8% of the mitochondria, 88 ± 1% of the endoplasmic reticulum, and 88 ± 1% of the plasma membrane. PSs are known to localize and accumulate in several cell organelles, a process which in turn depends on intrinsic characteristics, such as charge distribution, hydrophilicity/lipophilicity, shape, size, and overall structure. This is extremely important, given that it primarily determines the photosensitizer’s cellular uptake and subcellular localization, which will determine the PDT efficacy (Benov, 2015). In general, amphiphilic PSs bind to high-density lipoproteins (HDLs), hydrophobic ones mostly localize in the inner lipid core of low-density lipoproteins (LDLs), and hydrophilic photosensitizers bind customarily to albumin (Castano et al., 2005). Concerning the tissues, augmented lipophilicity generally contributes to higher uptake, while also influencing subcellular localization, as it moves from mainly concentrating in lysosomes toward mitochondria with increasing amphiphilicity (Pavani et al., 2009).

The PS subcellular localization is one of the factors determining the type of cell death by PDT. Chlorin **5a** mitochondrial targeting is particularly interesting since the disruption of mitochondrial functions may induce rapid apoptotic response. In fact, we have previously shown that the mitochondrial membrane potential is compromised after photodynamic treatment with chlorin **5a** in A375 cells (Pereira et al., 2015). On the other hand, photosensitizers targeting the endoplasmic reticulum have been reported to mediate necrosis (Benov, 2015). Moreover, the photosensitizers’ location on plasma membrane do not seem to favour apoptosis with caspase 3 inactivation (Kessel, 2004; Kim et al., 2014). Thus, the observed intracellular distribution agrees with the previously reported flow cytometry study carried out to determine the induced mechanism of cell death. After the photodynamic treatment of A375 melanoma cells with photosensitizer **5a**, apoptosis, and necrosis are involved in equal parts in the treatment response (Pereira et al., 2015).

## 3 Conclusion

Novel 4,5,6,7-tetrahydropyrazolo[1,5-*a*]pyridine-fused *meso*-tetraarylchlorins having different amphiphilic properties, bearing functionalities with varying degrees of polarity at the exocyclic ring (methyl ester, hydroxymethyl, and carboxylic acid moieties) and substituents of different chemical nature at the aryl groups, have been synthesized. Photophysical characterization and *in vitro* biological assessment of these macrocycles as PDT agents against melanoma, esophageal adenocarcinoma, and bladder carcinoma cell lines were carried out.

The ϕ_F_ values were found to remain constant for the studied ring-fused chlorins. However, the singlet oxygen sensitization quantum yields of representative ring-fused chlorin photosensitizers (diester, dialcohol, monoester/monoalcohol, and diacid chlorins) indicate that the presence of carboxylic acid or ester functionalities significantly increases the ^1^O_2_ photosensitization process.

After showing the high efficacy of di(hydroxymethyl)-substituted *meso*-tetraphenylchlorin as PDT agent against melanoma cells, herein the great potential of this photosensitizer was corroborated by the observed IC_50_ values of 63 and 73 nM against OE19 and HT1376, respectively. In the diacid and monoalcohol series, chlorins derived from *meso*-tetraphenylporphyrin proved to be the more efficient PDT agents, showing IC_50_ values of 68 and 344 nM against A375 cells, respectively. However, these compounds were less active against OE19 and HT1376 cells, the diacid chlorin with IC_50_ values still in the nano-molar range, whereas the monoalcohol chlorin shows significant higher IC_50_ values. The cytotoxicity in the absence of light was evaluated for all the PSs and no toxicity was observed in concentrations up to 10 µM.

The photocytotoxicity of the studied chlorins does not correspond to the capability to produce singlet oxygen, but it correlates with the cellular uptake values, where PSs with one or two methyl ester substituents present low cellular uptake values, PSs with two carboxylic acid substituents present intermediate cell uptake values, and PSs with two hydroxymethyl groups showing the higher internalization.

The lead di(hydroxymethyl)-substituted *meso*-tetraphenylchlorin remains the most promising PDT agent within these free-base ring-fused chlorin series. Thus, intracellular localization of this chlorin in A375 melanoma cells was studied to determine its initial targets in PDT. The lead chlorin seems to be unable to surpass the nuclear envelope, but subcellular accumulation in the mitochondria, endoplasmic reticulum, and plasma membrane was demonstrated. The observed intracellular distribution indicates that apoptosis and necrosis are the induced mechanism of cell death involved in the treatment response.

## 4 Experimental

### 4.1 Chemistry

#### 4.1.1 General

Commercially available high-grade materials and reagents were used as received. Organic solvents were purified by standard procedures prior to utilization (Armarego and Perrin, 1997). Microwave-assisted reactions were carried out with a CEM Discover S-Class-focused microwave reactor featuring continuous temperature, pressure, and microwave power control, under closed vessel conditions. Reaction monitoring was made by TLC analysis, on SiO_2_ 60 F254-coated aluminum plates, and *via* UV-vis absorption spectroscopy, using a PG Instruments T80, Hitachi U-2001, or Shimadzu UV-2100 spectrophotometer. Flash column chromatography was performed using SiO_2_ 60 (35–70 µm) as the stationary phase. Melting points were determined with a FALC R132467 electrothermal apparatus, using open glass capillaries, and are uncorrected. NMR spectra were recorded at room temperature with a Bruker Avance III spectrometer, operating at 400 MHz (^1^H) and 100 MHz (^13^C). Tetramethylsilane (TMS) was used as internal standard. Chemical shifts (*δ*) are expressed in parts per million related to TMS and coupling constants (*J*) are conveyed in hertz. HRMS spectra were obtained with a Waters Micromass VG Autospec M ESI-TOF spectrometer.

#### 4.1.2 Synthesis of Chlorin **4**

Chlorins **4** (Pereira et al., 2010; Pereira et al., 2011) were prepared from the reaction of 2,2-dioxo-1*H*,3*H*-pyrazolo[1,5-*c*][1,3]thiazole-6,7-dicarboxylate **1** (Sutcliffe et al., 2000; Sutcliffe et al., 2001) and *meso*-tetraarylporphyrin **3** (Liu et al., 2009) under microwave irradiation at 250°C for 20 min, as previously described.

#### 4.1.3 Synthesis of Chlorins **7**

The synthesis of chlorins **7** was performed based on a procedure described in the literature (Soai and Ookawa, 1986). To a stirred solution of the respective chlorin **4** (0.016–0.039 mmol) in THF (1 ml) was added lithium borohydride (6 equiv.), followed by the dropwise addition of methanol (0.25 ml). The reaction mixture was heated at 50°C and left stirring for 4 h under a saturated nitrogen atmosphere. Then, it was cooled with an ice bath and quenched by the addition of 2–3 drops of a 5% aqueous HCl solution and distilled water. The solvents were evaporated under reduced pressure and the products were purified by silica gel flash column chromatography, using ethyl acetate as eluent. Chlorins **7** were obtained as purple solids.

*Chlorin* ***7a*** (60% yield, 14.2 mg, 0.018 mmol) was obtained from **4a** (25 mg, 0.030 mmol) as described in the general procedure.

mp > 250°C. ^1^H NMR (400 MHz, CDCl_3_): δ 8.60 (d, AB system, *J* = 3.9 Hz, 1H, β-H pyrrolic), 8.59 (d, AB system, *J* = 3.9 Hz, 1H, β-H pyrrolic), 8.44 (s, 2H, β-H pyrrolic), 8.29 (d, *J* = 8.0 Hz, 2H, β-H pyrrolic), 8.23–8.17 (m, 4H, Ar), 8.09–8.03 (m, 3H, Ar), 7.96–7.94 (m, 1H, Ar), 786–7.81 (m, 2H, Ar), 7.76–7.66 (m, 10H, Ar), 5.70–5.63 (m, 1H, reduced β-H pyrrolic), 5.43–5.37 (m, 1H, reduced β-H pyrrolic), 4.58 (s, 2H, CH_2_OH), 4.22 (dd, *J* = 13.5, 7.6 Hz, 1H, CH_2_ from fused ring), 3.85 (dd, *J* = 13.5, 9.8 Hz, 1H, CH_2_ from fused ring), 3.75 (s, 3H, CO_2_Me), 3.56 (dd, *J* = 15.9, 6.9 Hz, 1H, CH_2_ from fused ring), 2.63 (dd, *J* = 15.9, 9.8 Hz, 1H, CH_2_ from fused ring), -1.63 (s, 2H, NH) ppm. ^13^C NMR (100 MHz, CDCl_3_): δ 165.3, 164.9, 162.3, 153.7, 153.1, 153.0, 144.2, 141.8, 141.7, 141.3, 141.1, 140.9, 133.9, 132.5, 132.4, 132,2, 128.8, 128.7, 128.5, 128.3, 127.8, 126.8, 124.4, 124.3, 112.6, 112.4, 107.6, 58.6, 51.4, 48.6, 48.4, 45.6, 26.3 ppm. HMRS (ESI): m/z = 797.3205 (found), 797.3235 calcd for [C_52_H_41_N_6_O_3_ (M + H)^+^].

*Chlorin* ***7b*** (25% yield, 4 mg, 0.004 mmol) was obtained from **4b** (15 mg, 0.016 mmol) as described in the general procedure.

mp > 250°C. ^1^H NMR (400 MHz, CDCl_3_): δ 8.62 (d, *J* = 5.0 Hz, 1H, β-H pyrrolic), 8.60 (d, *J* = 5.0 Hz, 1H, β-H pyrrolic), 8.45 (s, 2H, β-H pyrrolic), 8.31 (d, *J* = 4.9 Hz, 1H, β-H pyrrolic), 8.26 (d, *J* = 4.9 Hz, 1H, β-H pyrrolic), 8.12–8.02 (m, 6H, Ar), 7.96–7.94 (m, 1H, Ar), 7.85–7.82 (m, 1H, Ar), 7.37–7.27 (m, 4H, Ar), 7.22–7.17 (m, 4H, Ar), 5.67–5.60 (m, 1H, reduced β-H pyrrolic), 5.43–5.39 (m, 1H, reduced β-H pyrrolic), 4.61 (d, *J* = 2.5 Hz, 1H, CH_2_OH), 4.60 (d, *J* = 2.5 Hz, 1H, CH_2_OH), 4.34 (dd, *J* = 13.5, 7.4 Hz, 1H, CH_2_ from fused ring), 4.06 (m, 9H, OMe), 4.05 (m, 3H, OMe), 3.83 (dd, *J* = 13.5, 9.5 Hz, 1H, CH_2_ from fused ring), 3.77 (s, 3H, CO_2_Me), 3.59 (dd, *J* = 16.0, 7.1 Hz, 1H, CH_2_ from fused ring), 2.60 (dd, *J* = 16.0, 9.8 Hz, 1H, CH_2_ from fused ring), −1.61 (s, 2H, NH) ppm. ^13^C NMR (100 MHz, CDCl_3_): δ 165.4, 165.2, 162.4, 159.5, 159.4, 159.2, 153.6, 153.4, 153.2, 144.4, 141.4, 141.3, 136.3, 135.8, 135.6, 135.0, 134.2, 133.5, 132.9, 132.3, 132.2, 128.1, 124.2, 124.1, 123.2, 122.9, 113.9, 113.5, 112.9, 112.3, 111.8, 111.6, 107.6, 58.6, 55.5, 51.2, 48.7, 48.3, 45.5, 26.2 ppm. HMRS (ESI): m/z = 916.3562 (found), 917.3657 calcd for [C_56_H_49_N_6_O_7_ (M+H)^+^].

*Chlorin* ***7c*** (41% yield, 12 mg, 0.014 mmol) was obtained from **4c** (30 mg, 0.034 mmol) as described in the general procedure.

mp > 250°C. ^1^H NMR (400 MHz, CDCl_3_): δ 8.62 (d, AB system, *J* = 5.1 Hz, 1H, β-H pyrrolic), 8.61 (d, AB system, *J* = 5.1 Hz, 1H, β-H pyrrolic), 8.46 (s, 2H, β-H pyrrolic), 8.30–8.29 (m, 2H, β-H pyrrolic), 8.12–7.92 (m, 7H, Ar), 7.82–7.80 (m, 1H, Ar), 7.66–7.63 (m, 2H, Ar), 7.57–7.47 (m, 6H, Ar), 5.72–5.65 (m, 1H, reduced β-H pyrrolic), 5.41–5.34 (m, 1H, reduced β-H pyrrolic), 4.61 (s, 2H, CH_2_OH), 4.30 (dd, *J* = 13.4, 7.7 Hz, 1H, CH_2_ from fused ring), 3.87 (dd, *J* = 13.4, 9.5 Hz, 1H, CH_2_ from fused ring), 3.77 (s, 3H, CO_2_Me), 3.64 (dd, *J* = 16.0, 6.8 Hz, 1H, CH_2_ from fused ring), 2.67 (s, 12H, Me), 2.60 (dd, *J* = 16.0, 10.0 Hz, 1H, CH_2_ from fused ring), −1.62 (s, 2H), NH) ppm. ^13^C NMR (100 MHz, CDCl_3_): δ 165.5, 165.0, 162.5, 153.7, 153.3, 153.1, 144.5, 141.3, 141.1, 139.0, 138.4, 138.2, 137.5, 137.4, 135.8, 135.6, 135.2, 134.7, 134.4, 134.0, 132.5, 132.4, 132.1, 131.8, 129.6, 129.4, 129.1, 128.2, 127.6, 124.3, 124.2, 123.6, 123.3, 112.4, 112.2, 107.6, 58.7, 51.4, 48.8, 48.3, 45.6, 26.5, 21.8, 21.7, 21.6 ppm. HMRS (ESI): m/z = 853.3838 (found), 853.3861 calcd for [C_56_H_49_N_6_O_3_ (M + H)^+^].

*Chlorin* ***7d*** (44% yield, 16 mg, 0.017 mmol) was obtained from **4d** (38 mg, 0.039 mmol) as described in the general procedure.

mp > 250°C. ^1^H NMR (400 MHz, CDCl_3_): δ 8.61 (d, *J* = 4.9 Hz, 1H, β-H pyrrolic), 8.59 (d, *J* = 4.9 Hz, 1H, β-H pyrrolic), 8.42 (s, 2H, β-H pyrrolic), 8.31 (d, *J* = 5.1 Hz, 1H, β-H pyrrolic), 8.25 (d, *J* = 4.9 Hz, 1H, β-H pyrrolic), 8.18–8.14 (m, 2H, Ar), 8.10-8-04 (m, 2H, Ar), 7.99–7.97 (m, 3H, Ar), 7.89–7.84 (m, 3H, Ar), 7.73–7.67 (m, 6H, Ar), 5.64–5.58 (m, 1H, reduced β-H pyrrolic), 5.46–5.39 (m, 1H, reduced β-H pyrrolic), 4.59 (d, *J* = 4.4 Hz, 2H, CH_2_OH), 4.33 (dd, *J* = 13.4, 7.2 Hz, 1H, CH_2_ from fused ring), 3.86 (dd, *J* = 13.4, 9.4 Hz, 1H, CH_2_ from fused ring), 3.82 (s, 3H, CO_2_Me), 3.56 (dd, *J* = 15.9, 7.2 Hz, 1H, CH_2_ from fused ring), 2.71 (dd, *J* = 15.9, 9.2 Hz, 1H, CH_2_ from fused ring), −1.69 (s, 2H, NH) ppm. ^13^C NMR (100 MHz, CDCl_3_): δ 165.1, 165.0, 162.2, 153.8, 153.0, 152.9, 143.7, 141.0, 140.8, 140.0, 139.9, 139.8, 139.5, 136.4, 135.7, 135.5, 135.4, 135.3, 134.9, 134.8, 134.4, 134.3, 133.2, 133.1, 132.5, 132.4, 129.1, 129.0, 128.6, 128.3, 128.2, 127.9, 127.1, 124.4, 124.3, 122.4, 122.1, 111.4, 111.2, 107.8, 58.5, 51.6, 48.5, 48.3, 45.4, 26.2 ppm. HMRS (ESI): m/z = 933.1674 (found), 933.1676 calcd for [C_52_H_37_Cl_4_N_6_O_3_ (M + H)^+^].

#### 4.1.4 Synthesis of Chlorins **6**

To a stirred solution of the respective chlorin **4** (0.053–0.065 mmol) in THF (6 ml) saturated aqueous KOH solution (4 ml) was added, with the reaction mixture being stirred at room temperature for 24 h. After evaporation of the solvent under reduced pressure, the crude product mixture was placed in an ice bath and acidified by the careful addition of a 1 M aqueous HCl solution, followed by filtration of the resulting thin solids and thorough washing with distilled water until neutral pH. The products were purified by silica gel flash column chromatography, using dichloromethane/methanol (9:1) as eluent. Chlorins **6** were obtained as dark-purple solids.

*Chlorin* ***6a*** (67% yield, 33 mg, 0.041 mmol) was obtained from **4a** (51 mg, 0.062 mmol) as described in the general procedure.

mp > 250°C. ^1^H NMR (400 MHz, DMSO-d_6_): δ 8.57 (d, *J* = 4.9 Hz, 2H, β-H pyrrolic), 8.36–8.29 (m, 5H, 3xβ-H pyrrolic and 2xAr), 8.26 (d, *J* = 4.9 Hz, 1H, β-H pyrrolic), 8.22–8.16 (m, 2H, Ar), 8.12–7.94 (m, 5H, Ar), 7.90–7.68 (m, 11H, Ar), 5.81–5.74 (m, 1H, reduced β-H pyrrolic), 5.49–5.42 (m, 1H, reduced β-H pyrrolic), 4.26 (dd, *J* = 13.5, 6.4 Hz, 1H, CH_2_ from fused ring), 4.08 (dd, *J* = 13.5, 6.4 Hz, 1H, CH_2_ from fused ring), 3.51–3.40 (m, 4H, 2×CH_2_ from fused ring and 2×CO_2_H), −1.85 (s, 2H, NH) ppm. HRMS (ESI): m/z = 797.2836 (found), 797.2870 calcd for [C_51_H_37_N_6_O_4_ (M + H)^+^].

*Chlorin* ***6b*** (43% yield, 25 mg, 0.027 mmol) was obtained from **4b** (60 mg, 0.063 mmol) as described in the general procedure.

mp > 250°C. ^1^H NMR (400 MHz, DMSO-d_6_): δ 8.59 (d, AB system, *J* = 4.0 Hz, 1H, β-H pyrrolic), 8.58 (d, AB system, *J* = 4.0 Hz, 1H, β-H pyrrolic), 8.33 (s, 2H, β-H pyrrolic), 8.29 (d, *J* = 4.9 Hz, 1H, β-H pyrrolic), 8.26 (d, *J* = 4.9 Hz, 1H, β-H pyrrolic), 8.23 (dd, *J* = 8.4, 1.7 Hz, 1H, Ar), 8.18 (dd, *J* = 8.4, 1.7 Hz, 1H, Ar), 8.12–8.06 (m, 2H, Ar), 8.01–7.89 (m, 4H, Ar), 7.51 (dd, *J* = 8.4, 2.3 Hz, 1H, Ar), 7.42 (dd, *J* = 8.4, 2.3 Hz, 1H, Ar), 7.33–7.28 (m, 5H, Ar), 7.25 (dd, *J* = 8.4, 2.3 Hz, 1H, Ar), 5.78–5.72 (m, 1H, reduced β-H pyrrolic), 5.48–5.41 (m, 1H, reduced β-H pyrrolic), 4.29–4.22 (m, 1H, CH_2_ from fused ring), 4.17–4.10 (m, 1H, CH_2_ from fused ring), 4.04 (s, 3H, OMe), 4.02 (s, 3H, OMe), 4.01 (s, 6H, OMe), 3.49–3.38 (m, 4H, 2×CH_2_ from fused ring and 2×CO_2_H), −1.84 (s, 2H, NH) ppm. HRMS (ESI): m/z = 917.3291 (found), 917.3293 calcd for [C_55_H_45_N_6_O_8_ (M + H)^+^].

*Chlorin* ***6c*** (83% yield, 46 mg, 0.054 mmol) was obtained from **4c** (57 mg, 0.065 mmol) as described in the general procedure.

mp > 250°C. ^1^H NMR (400 MHz, DMSO-d_6_): δ 8.56 (d, AB system, *J* = 4.7 Hz, 1H, β-H pyrrolic), 8.55 (d, AB system, *J* = 4.7 Hz, 1H, β-H pyrrolic), 8.30 (s, 2H, β-H pyrrolic), 8.27 (d, AB system, *J* = 4.9 Hz, 1H, β-H pyrrolic), 8.25 (d, AB system, *J* = 4.9 Hz, 1H, β-H pyrrolic), 8.23 (d, *J* = 7.6 Hz, 1H, Ar), 8.16 (d, *J* = 7.6 Hz, 1H, Ar), 8.05–8.00 (m, 2H, Ar), 7.90–7.82 (m, 4H, Ar), 7.73 (d, *J* = 7.6 Hz, 1H, Ar), 7.67 (d, *J* = 7.6 Hz, 1H, Ar), 7.57–7.45 (m, 6H, Ar), 5.76–5.71 (m, 1H, reduced β-H pyrrolic), 5.47–5.41 (m, 1H, reduced β-H pyrrolic), 4.27 (dd, *J* = 13.4, 6.2 Hz, 1H, CH_2_ from fused ring), 4.13 (dd, *J* = 13.4, 6.2 Hz, 1H, CH_2_ from fused ring), 3.44-3-42 (m, 2H, CH_2_ from fused ring), 2.62 (s, 3H, Me), 2.60 (s, 3H, Me), 2.59 (s, 6H, Me), −1.86 (s, 2H, NH) ppm. HRMS (ESI): m/z = 853.3495 (found), 853.3496 calcd for [C_55_H_45_N_6_O_4_ (M + H)^+^].

*Chlorin* ***6d*** (24% yield, 12 mg, 0.013 mmol) was obtained from **4d** (51 mg, 0.053 mmol) as described in the general procedure.

mp > 250°C. ^1^H NMR (400 MHz, DMSO-d_6_): δ 8.60 (d, AB system, *J* = 4.1 Hz, 1H, β-H pyrrolic), 8.59 (d, AB system, *J* = 4.1 Hz, 1H, β-H pyrrolic), 8.37 (dd, *J* = 8.0, 1.2 Hz, 1H, Ar), 8.34–8.30 (m, 4H, 3×β-H pyrrolic and 1×Ar), 8.28 (d, *J* = 5.2 Hz, 1H, β-H pyrrolic), 8.22–8.18 (m, 2H, Ar), 8.11 (d, *J* = 8.0 Hz, 1H, Ar), 8.04–8.02 (m, 4H, Ar), 7.93 (dd, *J* = 8.0, 1.2 Hz, 1H, Ar), 7.86–7.75 (m, 6H, Ar), 5.79–5.73 (m, 1H, reduced β-H pyrrolic), 5.51–5.45 (m, 1H, reduced β-H pyrrolic), 4.32–4.24 (m, 1H, CH_2_ from fused ring), 4.18–4.13 (m, 1H, CH_2_ from fused ring), 3.50 (s, 2H, CO_2_H), 3.47–3.42 (m, 2H, CH_2_ from fused ring), −1.91 (s, 2H, NH) ppm. HRMS (ESI): m/z = 933.1299 (found), 933.1311 calcd for [C_51_H_33_Cl_4_N_6_O_4_ (M + H)^+^].

### 4.2 Photophysics

Solvents were of spectroscopic grade and used as received. Absorption and fluorescence emission spectra were recorded on Cary 5000 UV-Vis-NIR and Horiba-Jobin-Ivon Fluorolog 322 spectrometers, respectively. All the fluorescence emission spectra were corrected for the wavelength response of the system. Room temperature fluorescence quantum yields were obtained by the comparative method using tetraphenylporphyrin (TPP) in toluene (ϕ_F_ = 0.11, as reference compound (Montalti et al., 2006). Singlet oxygen quantum yields, ϕ_Δ_, were determined by the direct measurement of the phosphorescence at 1,270 nm, followed by the irradiation of the aerated solution of the samples in DMSO with the excitation at 355 nm from a Nd:YAG laser with a setup elsewhere described (Seixas de Melo et al., 2013). TPP in toluene was used as standard (φ_Δ_ = 0.66) (Pineiro et al., 1998). For chlorin **4a** the fluorescence quantum yield and singlet oxygen sensitization quantum yield were obtained using **5a** as the reference compound. In this case, the ϕ_Δ_ value was obtained comparing the characteristic steady-state phosphorescence emission spectra of singlet oxygen, photosensitized by chlorins **4a** and **5a,** λ_exc_ = 424 nm, in aerated DMSO solutions.

### 4.3 Cell Biology

#### 4.3.1 Cell Culture Conditions

Human A375 (CRL1619) skin malignant melanoma and HT1376 (CRL1472) urinary bladder carcinoma cell lines were purchased from the American Type Culture Collection. Human OE19 (96071721) esophageal adenocarcinoma cell line was purchased from the European Collection of Authenticated Cell Cultures. All cell lines were cultured according to standard procedures, at 37°C, in a humidified incubator with 95% air and 5% CO_2_. A375 and HT1376 cell lines were expanded using the Dulbecco’s Modified Eagle medium (DMEM, Sigma D-5648), supplemented with 10% heat-inactivated fetal bovine serum (FBS, Sigma F7524), 1% Penicillin–Streptomycin (100 U/mL penicillin and 10 mg/ml streptomycin, Gibco 15,140-122), and 100 mM sodium pyruvate (Gibco Invitrogen Life Technologies; Gibco 1,360). The OE19 cell line was expanded using the Roswell Park Memorial Institute 1,640 media (RPMI 1640, Sigma R4130), supplemented with 10% heat-inactivated fetal bovine serum (FBS, Sigma F7524), 1% Penicillin–Streptomycin (100 U/mL penicillin and 10 mg/ml streptomycin, Gibco 15,140-122), and 400 mM sodium pyruvate (Gibco Invitrogen Life Technologies; Gibco 1,360). For all the studies, cells were detached using a solution of 0.25% trypsin-EDTA (Gibco).

#### 4.3.2 Photodynamic Treatment

For each experiment, cells were plated and kept in the incubator overnight, to allow the attachment of the cells. The formulation of the sensitizers consisted in a 1 mg/ml solution in DMSO (Fisher Chemical, 200-664-3), the desired concentrations being achieved by successive dilutions. The sensitizers were administered in several concentrations (from 1 nM to 10 µM) and cells were incubated for 24 h. Controls were included on every plate, including untreated cell cultures and cultures treated only with the vehicle of administration of the sensitizers. For this, DMSO was always administered with a concentration of 1% in the cell culture media. Cells were washed with phosphate buffered saline (PBS; in mM: 137NaCl (JMGS), 2.7 KCl (Sigma), 10 Na_2_HPO_4_ (Merck), and 1.8 KH_2_PO_4_ (Sigma), pH 7.4) and new drug-free medium was added. Each plate was irradiated with a flow rate of 7.5 mW/cm^2^ until a total of 10 J was reached using a light source equipped with a red filter (cut off <560 nm). Evaluation was performed 24 h after photodynamic treatment.

#### 4.3.3 Photocytotoxicity and Cytotoxicity

The sensitivity of the cell lines to the sensitizers was analyzed using the MTT colorimetric assay (Sigma M2128; Sigma-Aldrich, Inc.) to measure metabolic activity. Cell culture plates were washed and incubated with a solution of 3-(4,5-dimethylthiazol-2-yl)-2,5-diphenyltetrazolium bromide (0.5 mg/ml, Sigma M5655) in PBS, pH 7.4, in the dark at 37°C for at least 4 h. To solubilize formazan crystals, a 0.04 M solution of hydrochloric acid (Merck Millipore100317) in isopropanol (Sigma 278475) was added. Absorbance was measured using an EnSpire Multimode Plate Reader (Perkin Elmer). Cytotoxicity was expressed as the percentage relative to cell cultures treated only with the administration vehicle of the sensitizers. Dose-response curves were obtained using Origin 9.0 and the concentration of sensitizers that inhibits the proliferation of cultures in 50% (IC_50_) was derived. Dark cytotoxicity studies were performed as previously described but omitting the irradiation step.

#### 4.3.4 Cellular Uptake

Cells (5 × 10^5^) were incubated with sensitizers in concentrations of 500 nM during 24 h. Cells were then washed with PBS and disrupted with DMSO. Cell scrappers were used to ensure full disaggregation. The solutions were collected and centrifuged, the fluorescence intensity of the supernatants being determined by fluorescence emission spectroscopy with an EnSpire Multimode Plate Reader (Perkin Elmer), using 420 nm as the excitation wavelength. The intracellular concentration was determined using a calibration curve obtained from the fluorescence intensity in DMSO solutions for each sensitizer.

#### 4.3.5 Confocal Microscopy

Colocalization of chlorin **5a** in the nucleus, mitochondria, endoplasmic reticulum, and plasma membrane was evaluated through confocal microscopy using DAPI (4′,6-diamidino-2-phenylindole, dihydrochloride, Invitrogen™, D1306), MitoTracker® Green FM (Invitrogen™, M7514), ER-Tracker TM Green (BODIPY® FL glibenclamide, Invitrogen™, E34251), and CellMask™ Green plasma membrane stain (Invitrogen™, C37608), respectively. Briefly, 24-well plates with sterilized coverslips were used and the cells were incubated with 500 nM of chlorin **5a** for 24 h. The organelles were stained with the abovementioned probes, following the manufacturers’ recommendations, and the slides were prepared with ProLong™ Gold Antifade Mountant with DAPI (Invitrogen™, P36931). Image acquisition was performed on a Zeiss LSM 710 laser-scanning confocal microscope (Carl Zeiss, Germany) with an Axio Observer. Z1 component, using a ×40 oil objective (EC Plan-Neofluar ×40/1.30 Oil DIC) and the Zen Black 2010 software. At least 10 photographs of random fields of each coverslip were acquired in at least 2 independent experiments. Chlorin **5a** co-localization was analyzed with the ImageJ Fiji software, using a colocalization plugin, Coloc 2, through the Pearson’s correlation coefficient. For all images with the Pearson’s correlation coefficient greater than 0, the Mander’s colocalization indexes, tM2, were presented. tM2 corresponds to the colocalization of chlorin **5a** pixels colocalized with the labeled organelle pixels. The results were presented as the mean and the standard deviation of chlorin **5a** colocalization with each labeled organelle.

## Data Availability

The original contributions presented in the study are included in the article/Supplementary Material, further inquiries can be directed to the corresponding author.
