# Supplementary material for: Ring-Fused meso-Tetraarylchlorins as Auspicious PDT Sensitizers: Synthesis, Structural Characterization, Photophysics, and Biological Evaluation
Source: Front Chem. 2022 Apr 27;10:873245. doi: 10.3389/fchem.2022.873245 (PMC9091369; doi:10.3389/fchem.2022.873245)
Supplement: Supplementary file 1 [file DataSheet1.pdf]

**Ring-fused *meso*-Tetraarylchlorins as Auspicious PDT Sensitizers: Synthesis, Structural Characterization, Photophysics and Biological evaluation**

Mafalda Laranjo,[1,2,3] Nelson A.M. Pereira,[4] Andreia S.R. Oliveira,[4] Márcia Campos Aguiar,[1,4] Gonçalo Brites,[1] Bruno F.O. Nascimento,[4] Beatriz Serambeque[1,2], Bruna D.P. Costa[4], João Pina,[4] J. Sérgio Seixas de Melo,[4] Marta Pineiro,[4] M. Filomena Botelho,[1,2,3] Teresa M.V.D. Pinho e Melo[4*]

*[1]Institute of Biophysics and Institute for Clinical and Biomedical Research (iCBR), area of Environment Genetics and Oncobiology (CIMAGO), Faculty of Medicine, University of Coimbra, 3004-548 Coimbra, Portugal*
*[2]Centre of Innovative Biomedicine and Biotechnology (CIBB), University of Coimbra, 3004-548 Coimbra, Portugal*
*[3]Clinical and Academic Centre of Coimbra (CACC), 3004-548 Coimbra, Portugal*
*[4] University of Coimbra, Coimbra Chemistry Centre-Institute of Molecular Sciences and Department of Chemistry, 3004-535 Coimbra, Portugal*

**tmelo@ci.uc.pt*

**Supporting Information**

## Table of Contents

$^{1}$H and $^{13}$C NMR spectra of chlorins **6** and **7** **S2**

Mass spectra of chlorins **6** and **7** **S9**

Cytotoxicity of chlorin **6b** in A375 skin malignant melanoma cells, HT1376 urinary bladder carcinoma cells and OE19 esophageal adenocarcinoma cells **S17**

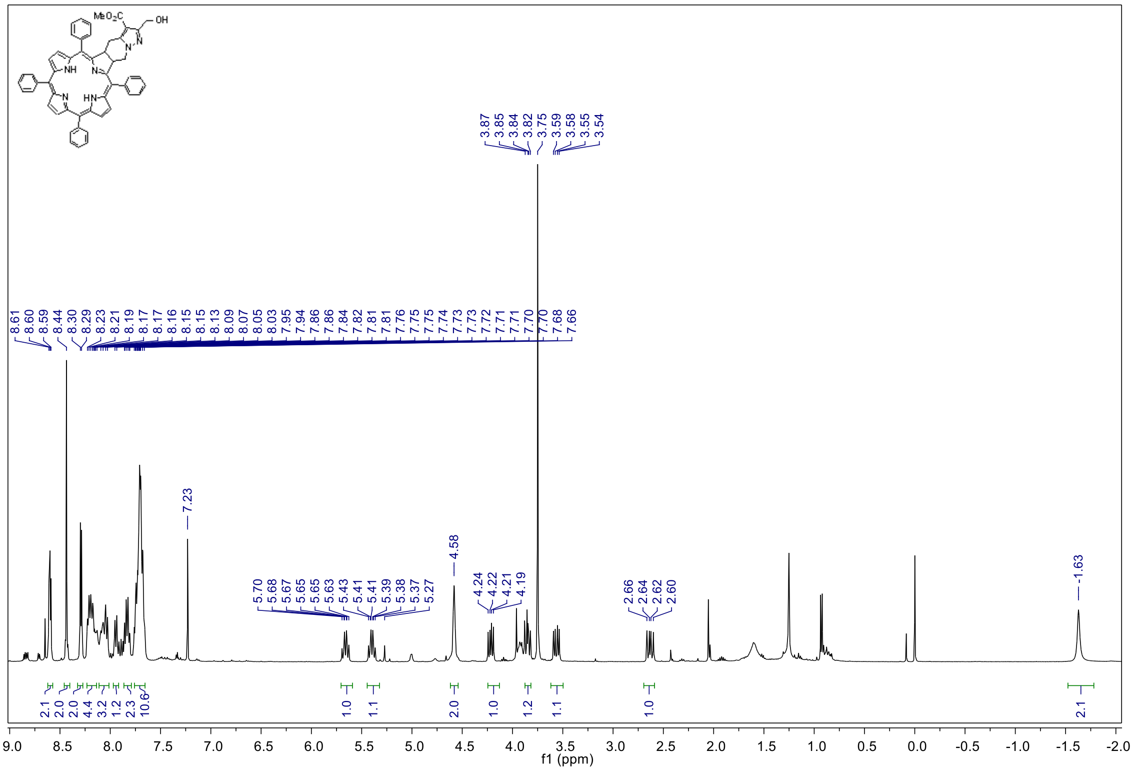

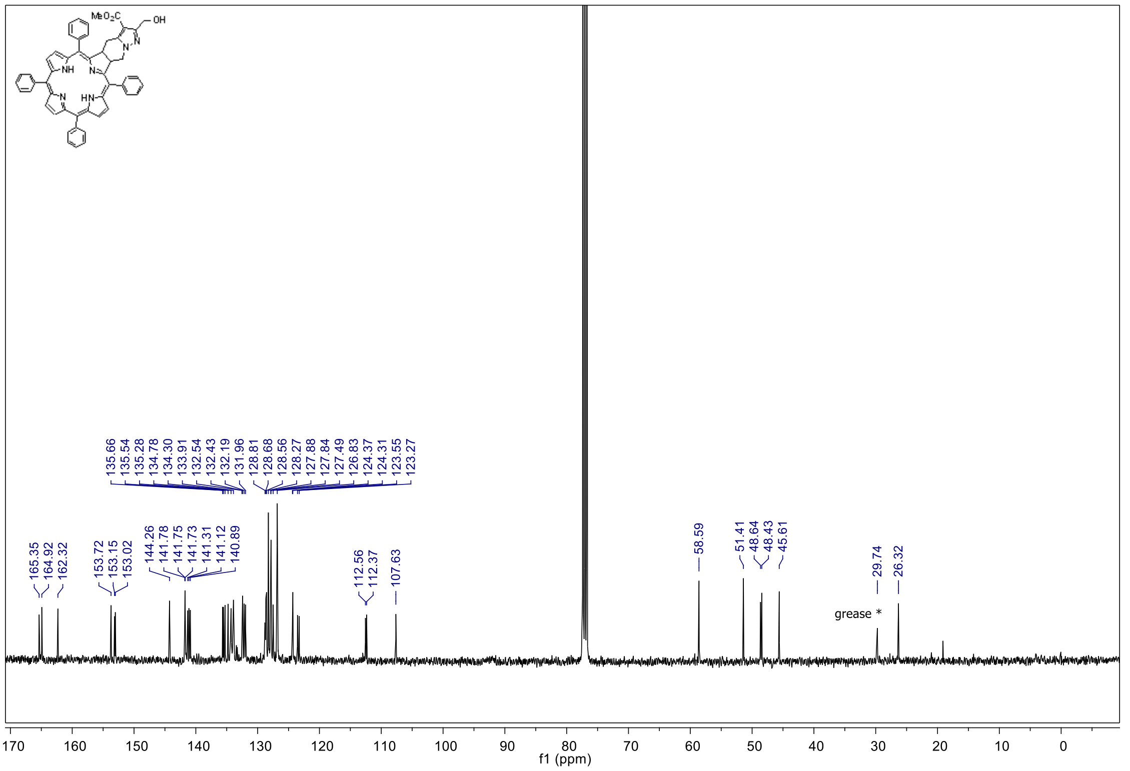

**Figure S1.** $^{1}H$ and $^{13}C$ NMR spectra ($CDCl_3$) of chlorin **7a**. Chemical shifts (δ) are given in ppm relative to internal TMS.

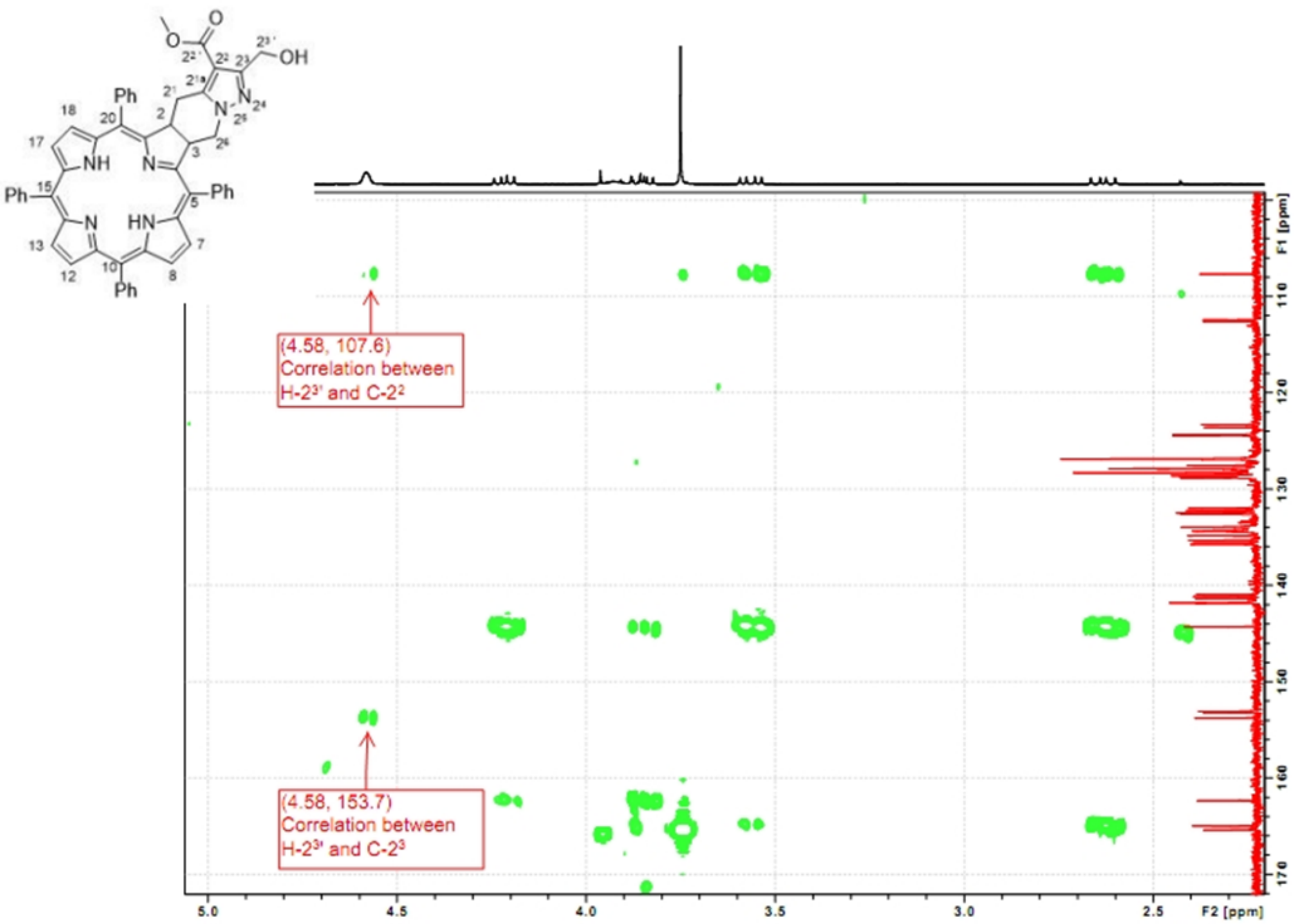


**Figure S2.** $^{1}H$-$^{13}C$ HMBC NMR spectrum ($CDCl_3$) of chlorin **7a**. Chemical shifts (δ) are given in ppm relative to internal TMS.

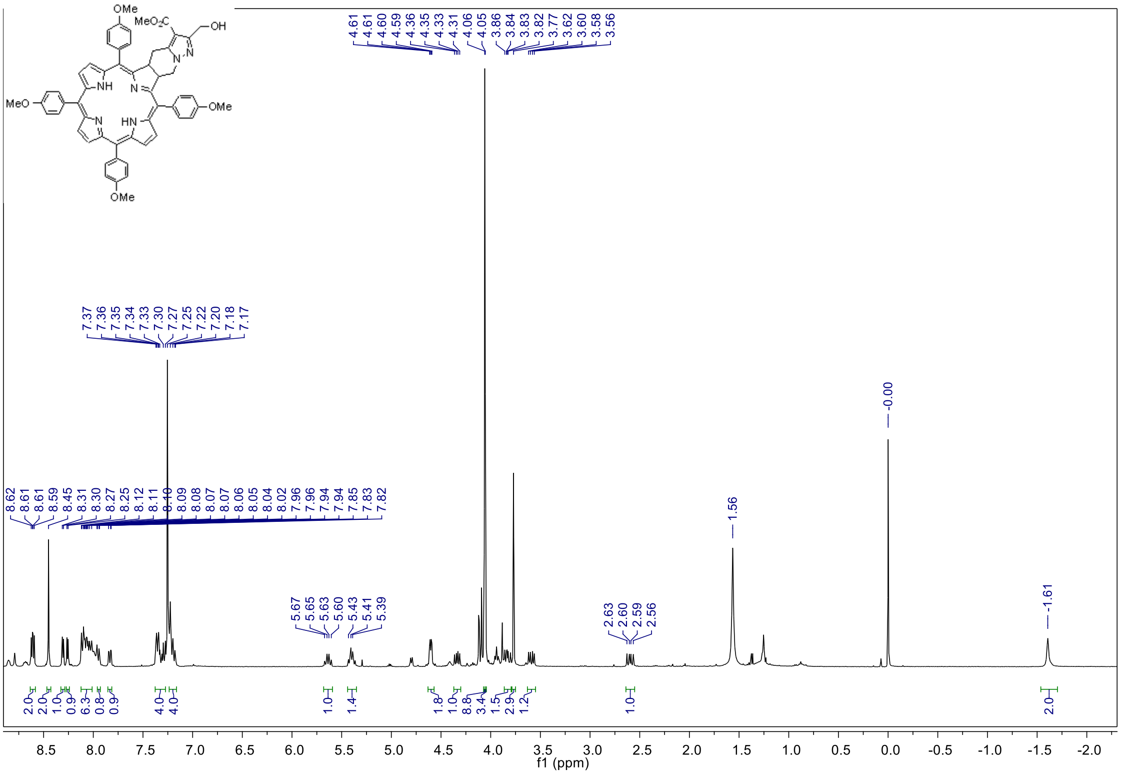


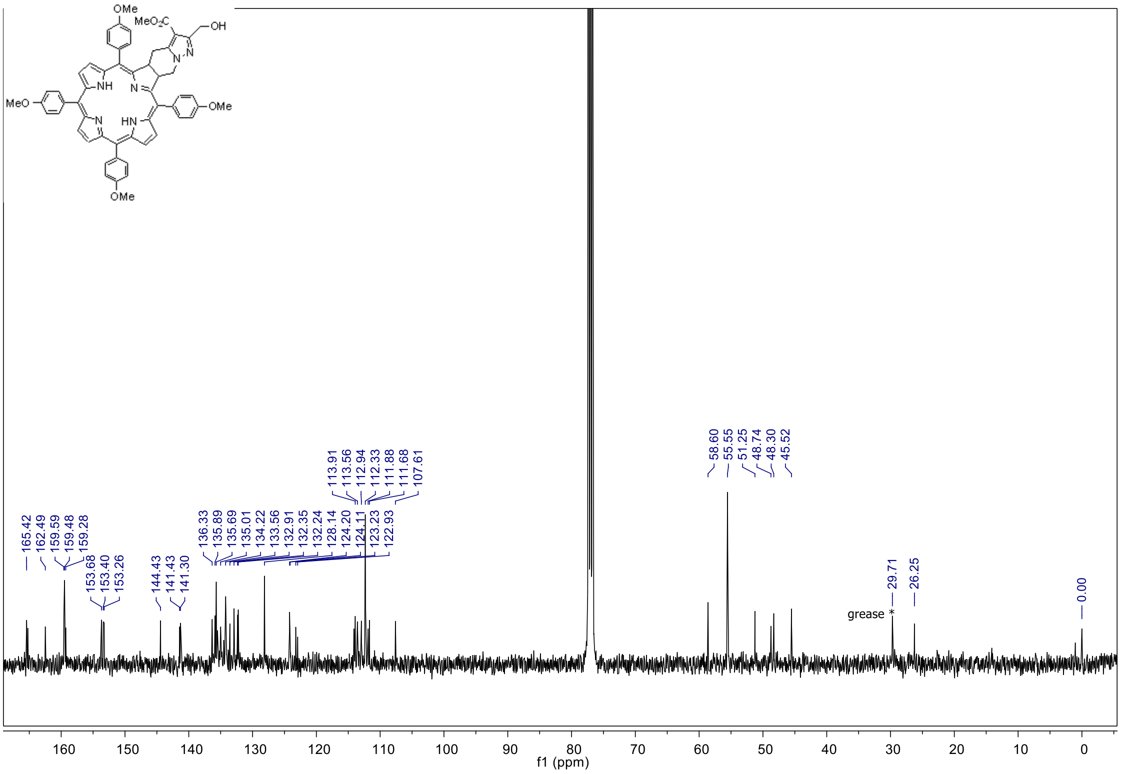


**Figure S3.** $^{1}H$ and $^{13}C$ NMR spectra ($CDCl_3$) of chlorin **7b**. Chemical shifts (δ) are given in ppm relative to internal TMS.

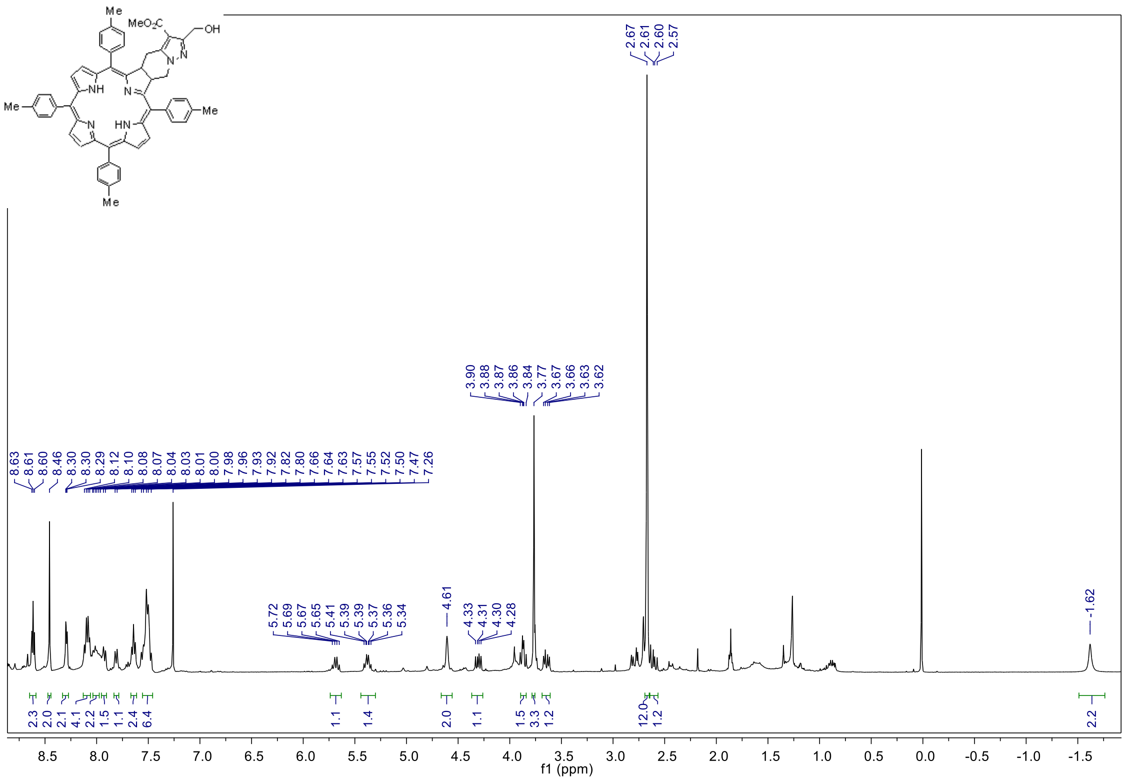

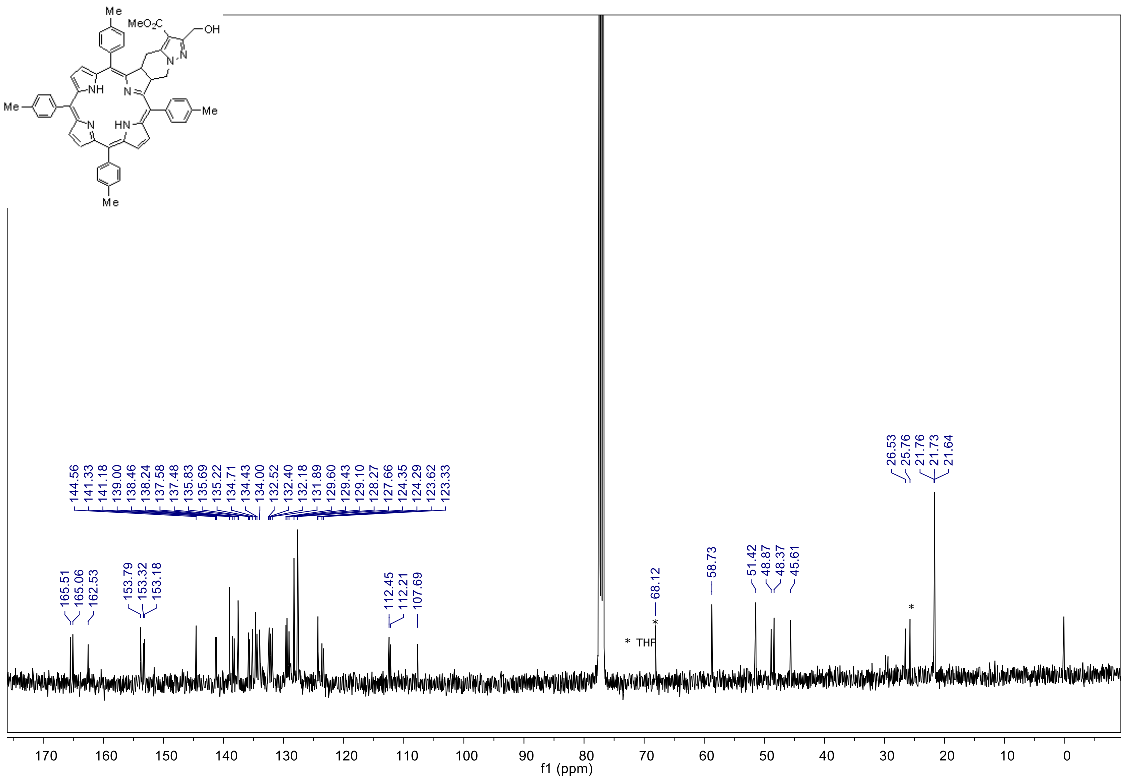

**Figure S4.** $^{1}H$ and $^{13}C$ NMR spectra ($CDCl_3$) of chlorin **7c**. Chemical shifts (δ) are given in ppm relative to internal TMS.

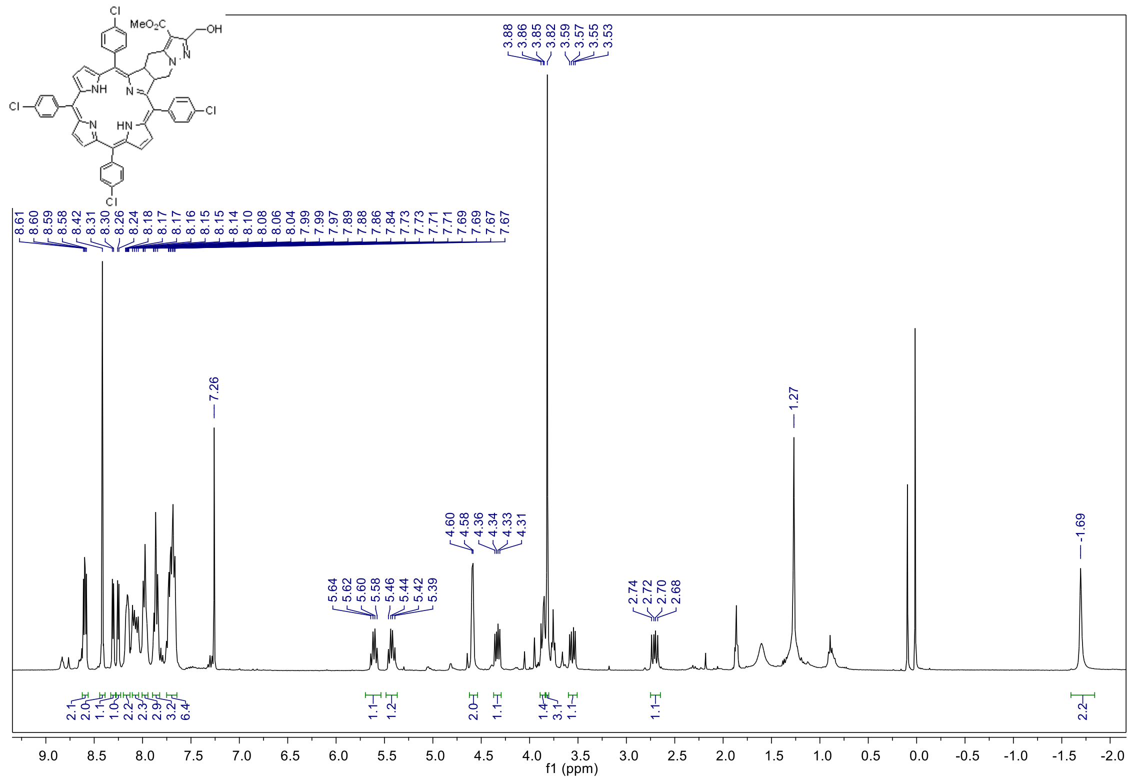

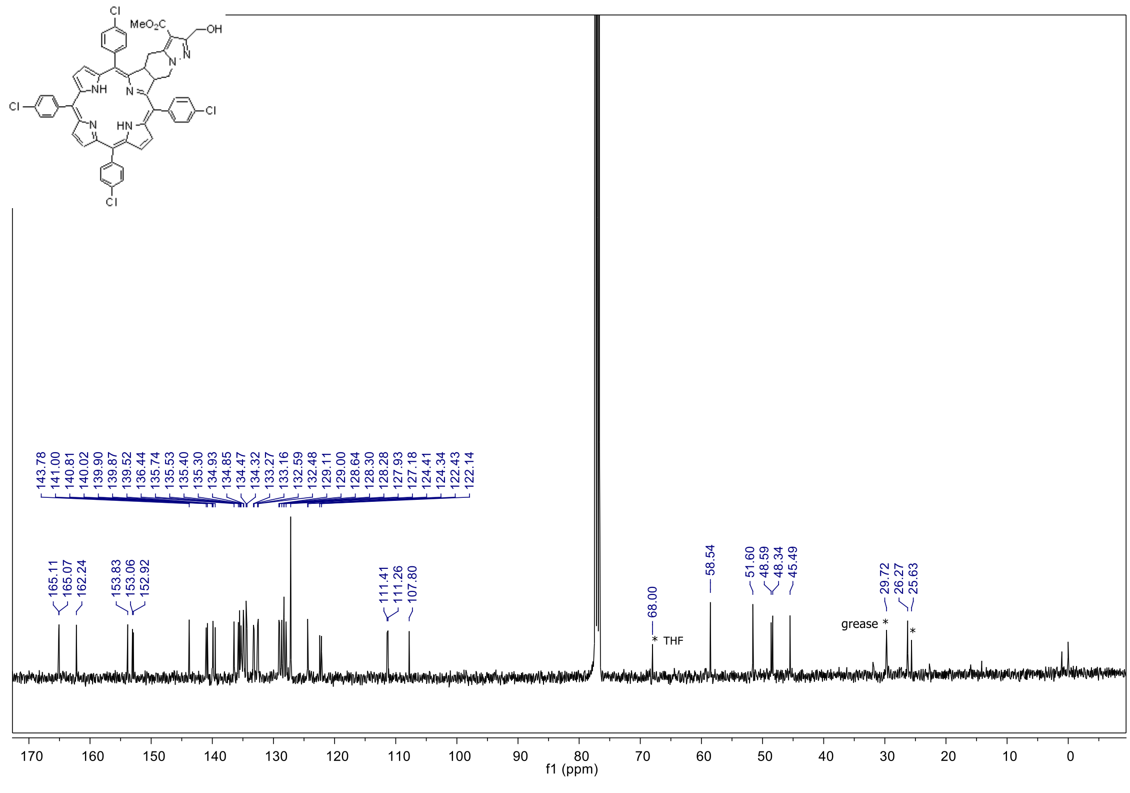

**Figure S5.** $^{1}H$ and $^{13}C$ NMR spectra ($CDCl_3$) of chlorin **7d**. Chemical shifts (δ) are given in ppm relative to internal TMS.

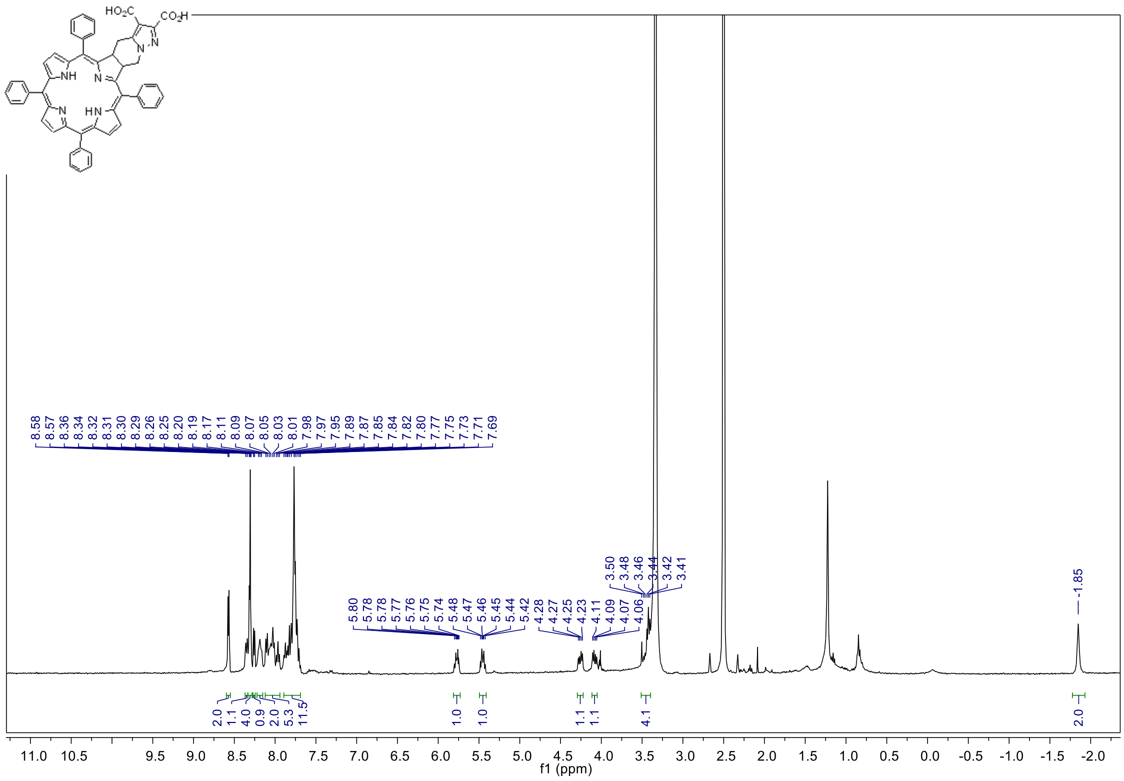

**Figure S6.** $^{1}H$ NMR spectrum (DMSO-$d_6$) of chlorin **6a**. Chemical shifts (δ) are given in ppm relative to solvent peak (2.50 ppm).

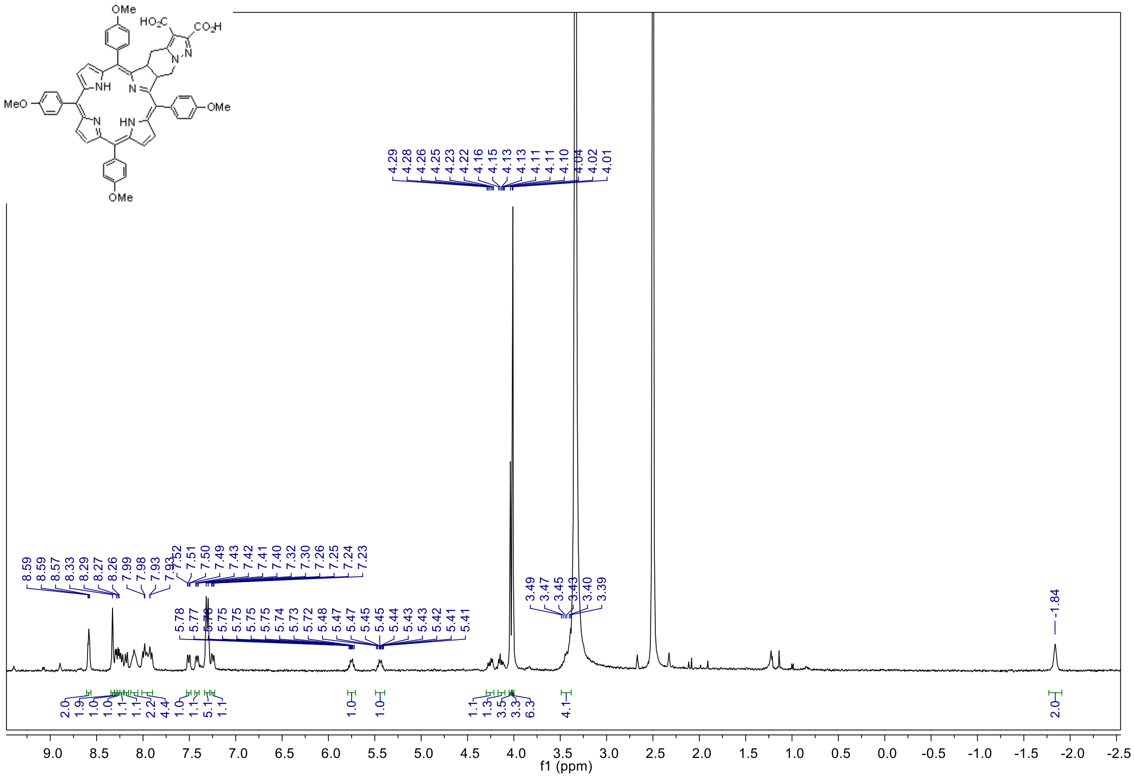

**Figure S7.** $^{1}H$ NMR spectrum (DMSO-$d_6$) of chlorin **6b**. Chemical shifts (δ) are given in ppm relative to solvent peak (2.50 ppm).

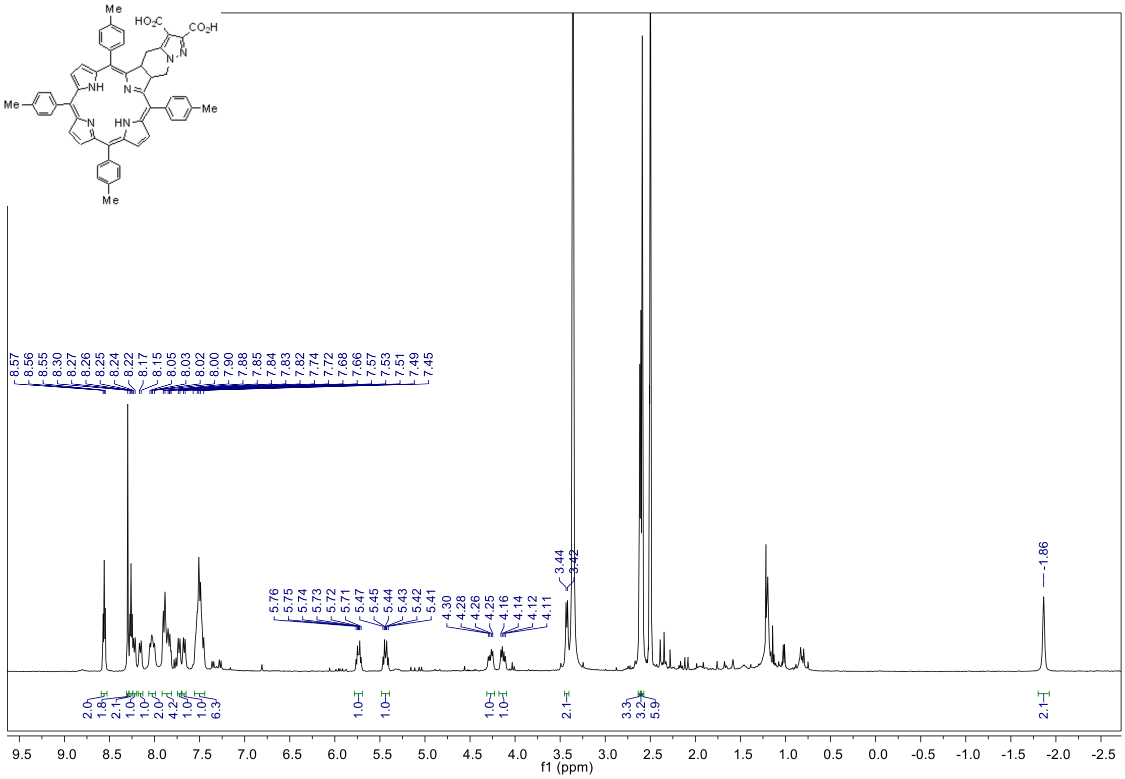


**Figure S8.** $^{1}$H NMR spectrum (DMSO-$d_6$) of chlorin **6c**. Chemical shifts (δ) are given in ppm relative to solvent peak (2.50 ppm).

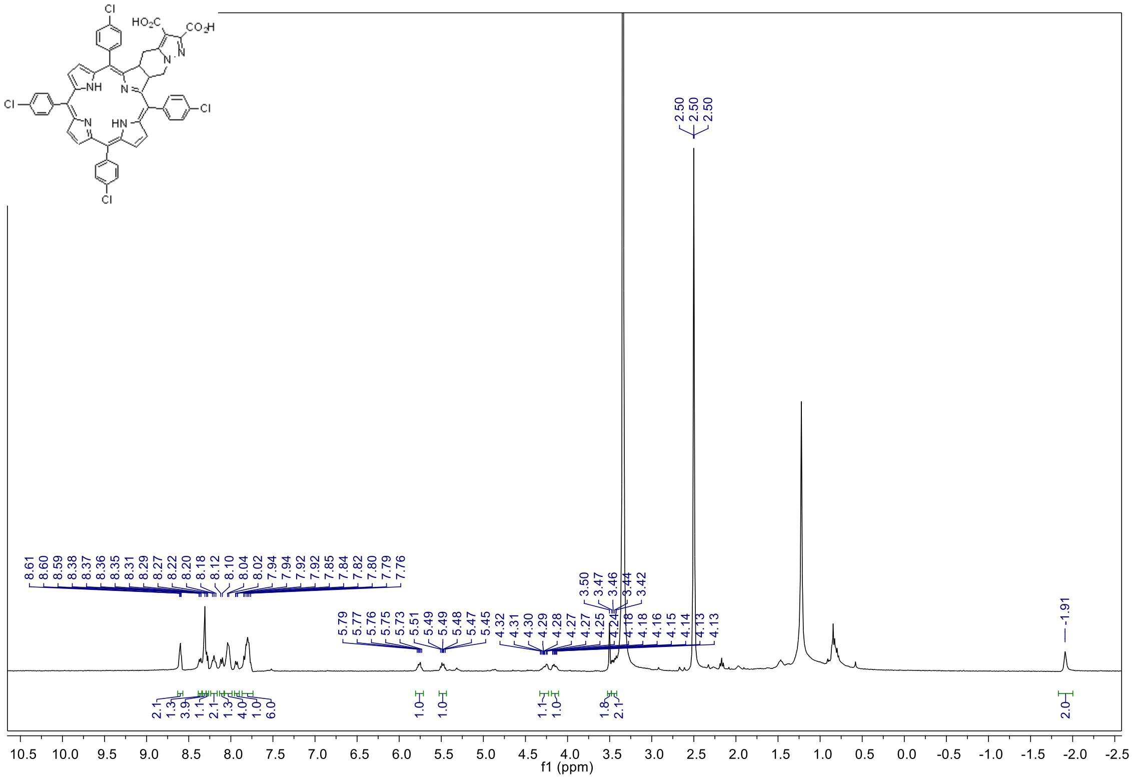


**Figure S9.** $^{1}$H NMR spectrum (DMSO-$d_6$) of chlorin **6d**. Chemical shifts (δ) are given in ppm relative to solvent peak (2.50 ppm).

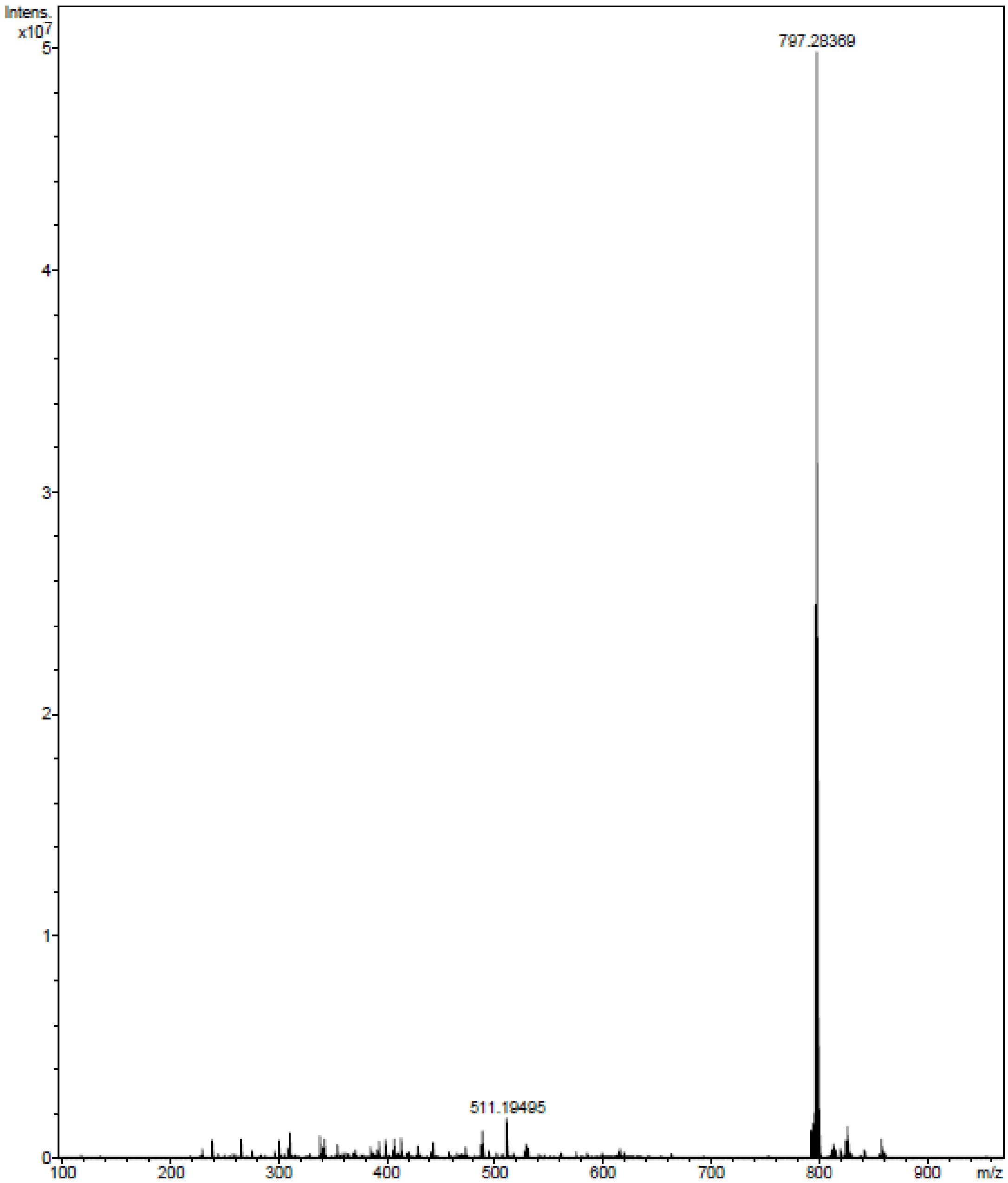


**Figure S10.** Electrospray ionization mass spectrum in positive-ion mode (HRMS-ESI+) of chlorin **6a**.

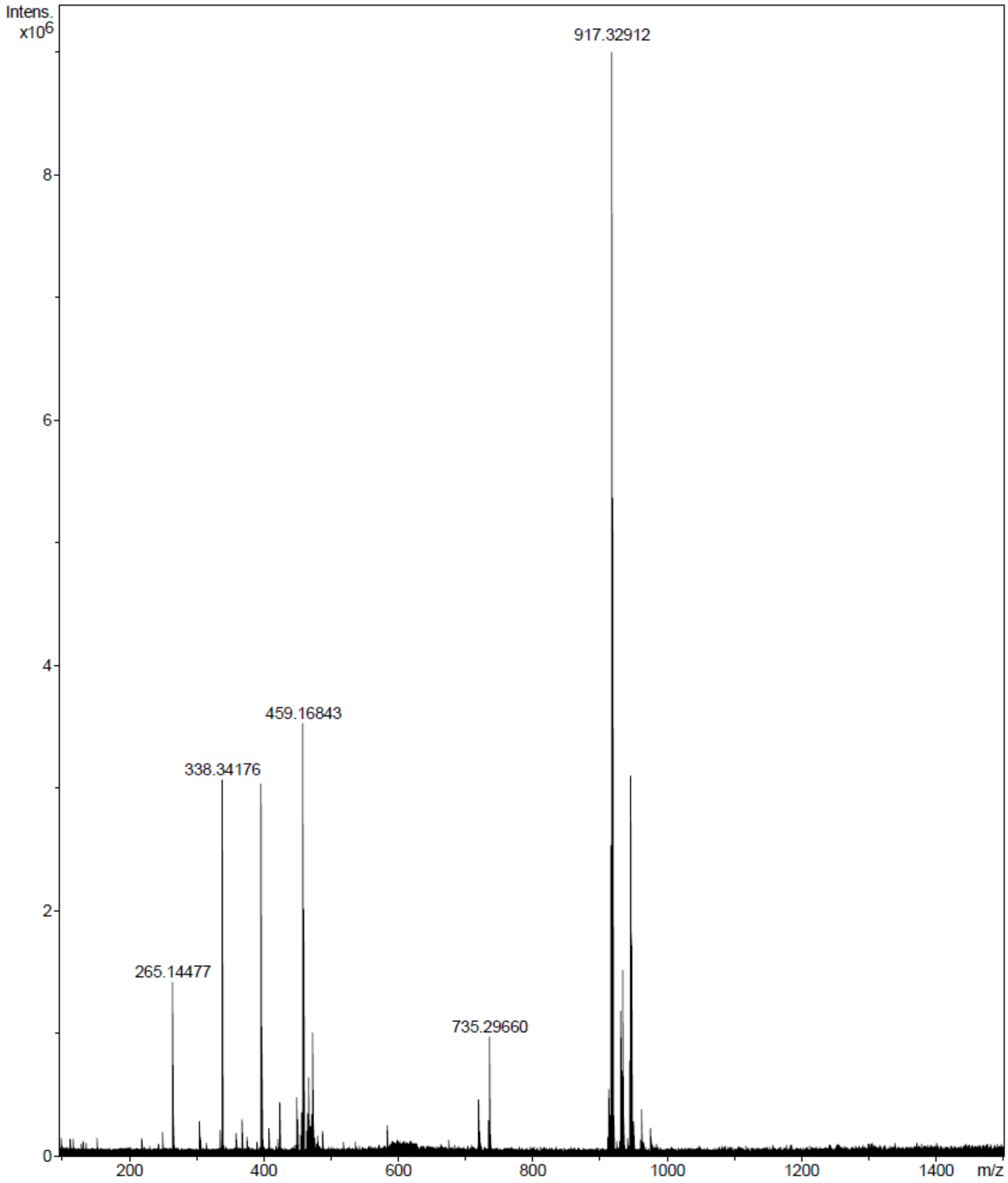


**Figure S11.** Electrospray ionization mass spectrum in positive-ion mode (HRMS-ESI+) of chlorin **6b**.

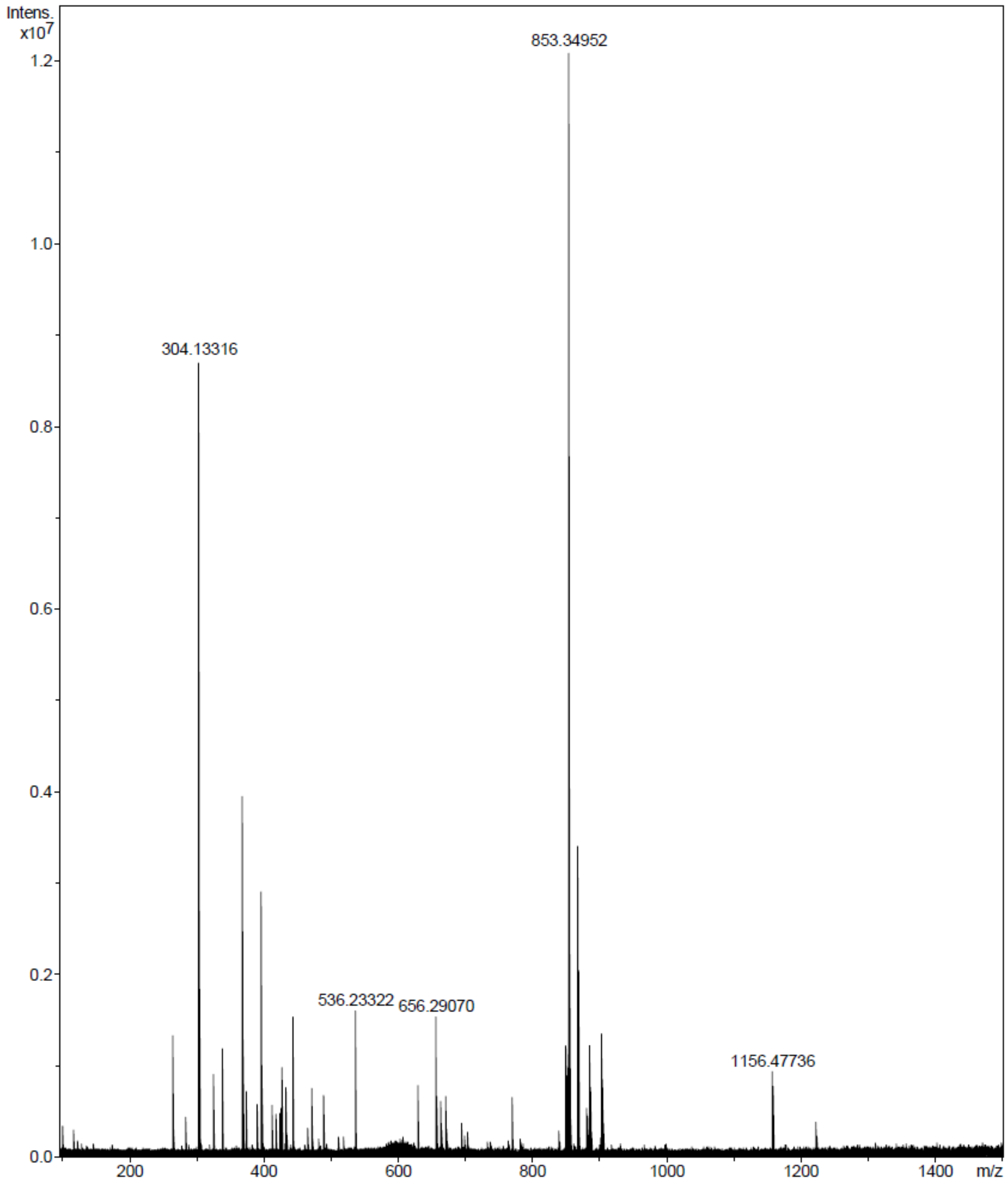


**Figure S12.** Electrospray ionization mass spectrum in positive-ion mode (HRMS-ESI+) of chlorin **6c**.

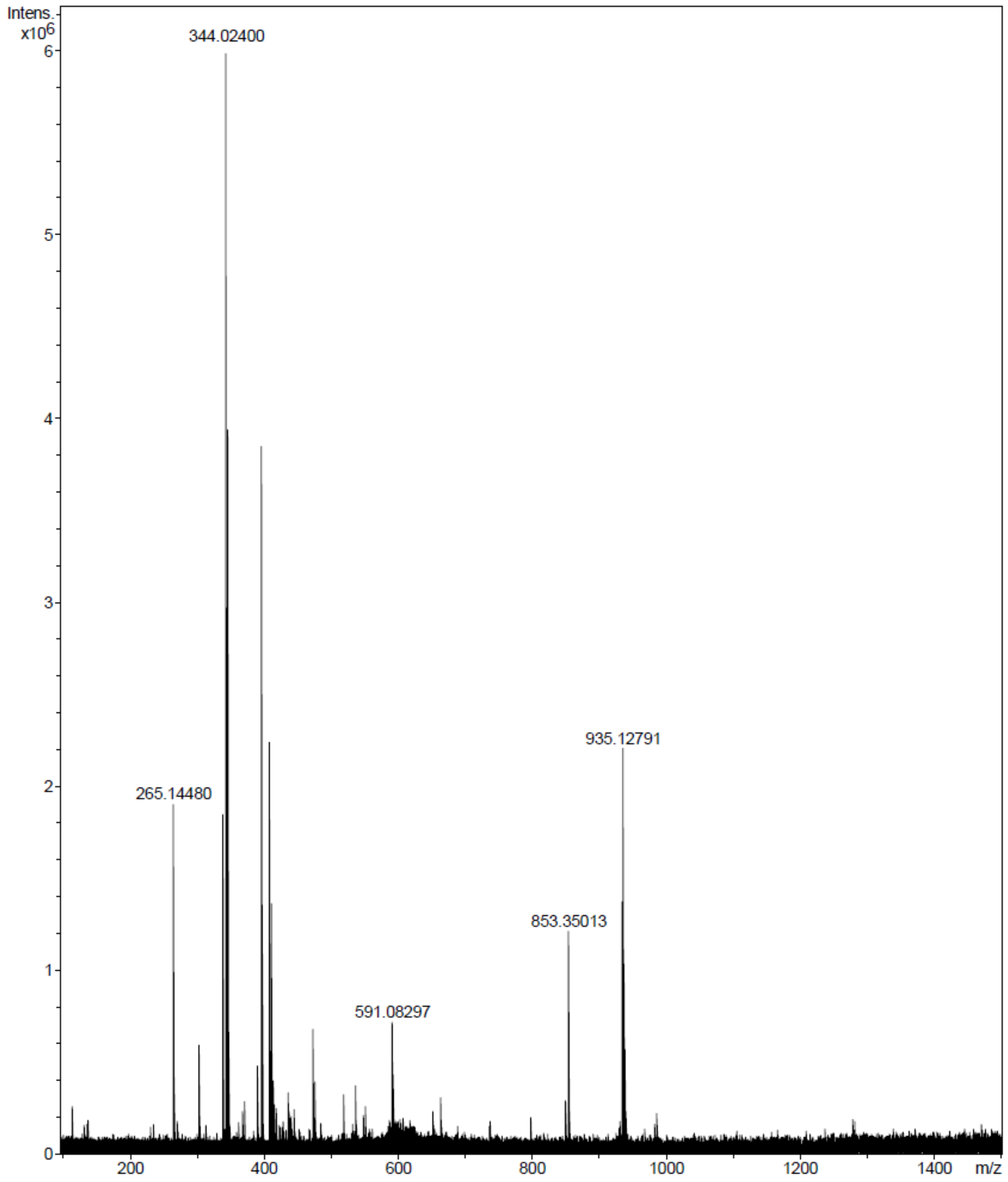


**Figure S13.** Electrospray ionization mass spectrum in positive-ion mode (HRMS-ESI+) of chlorin **6d**.

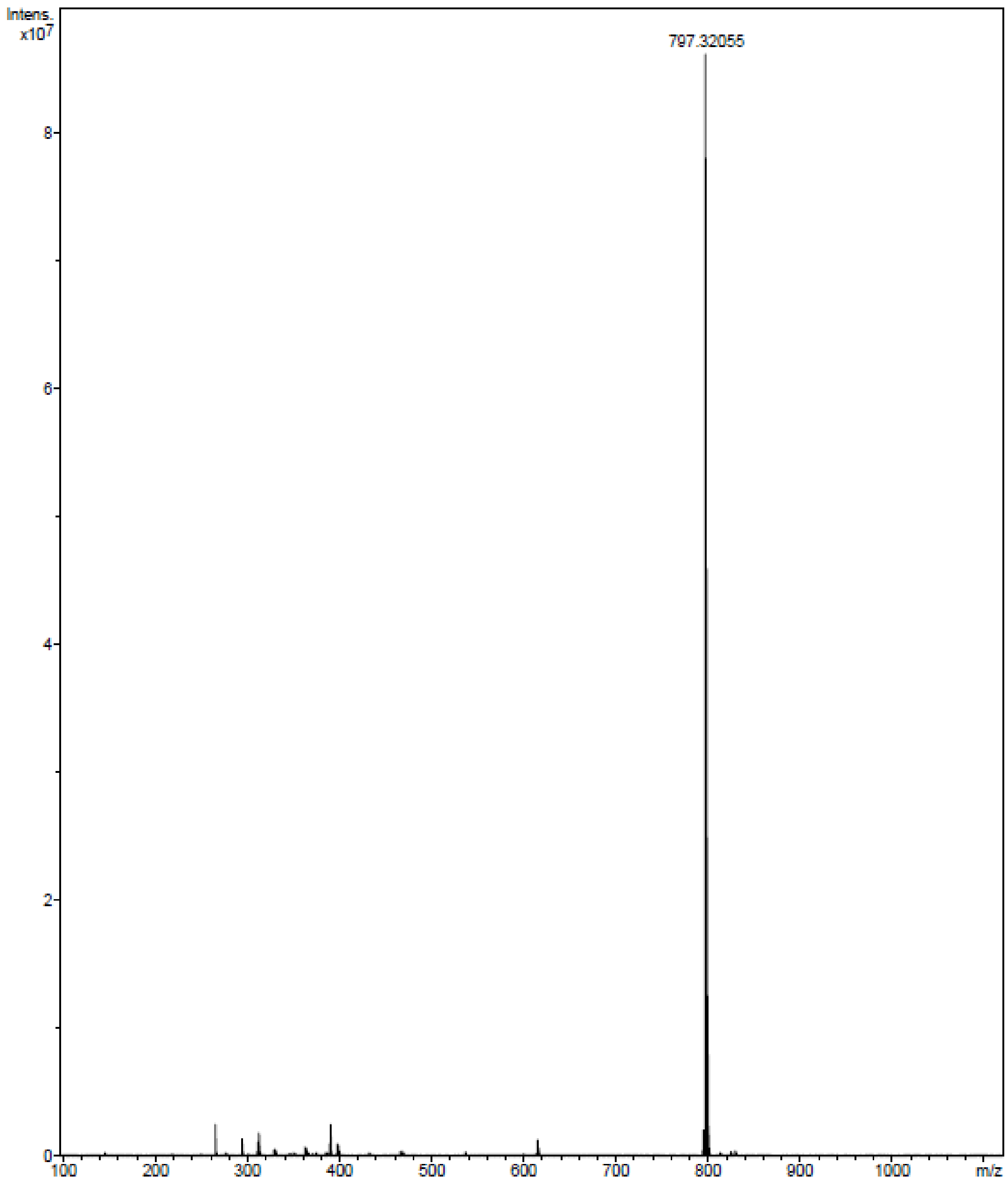


**Figure S14.** Electrospray ionization mass spectrum in positive-ion mode (HRMS-ESI+) of chlorin **7a**.

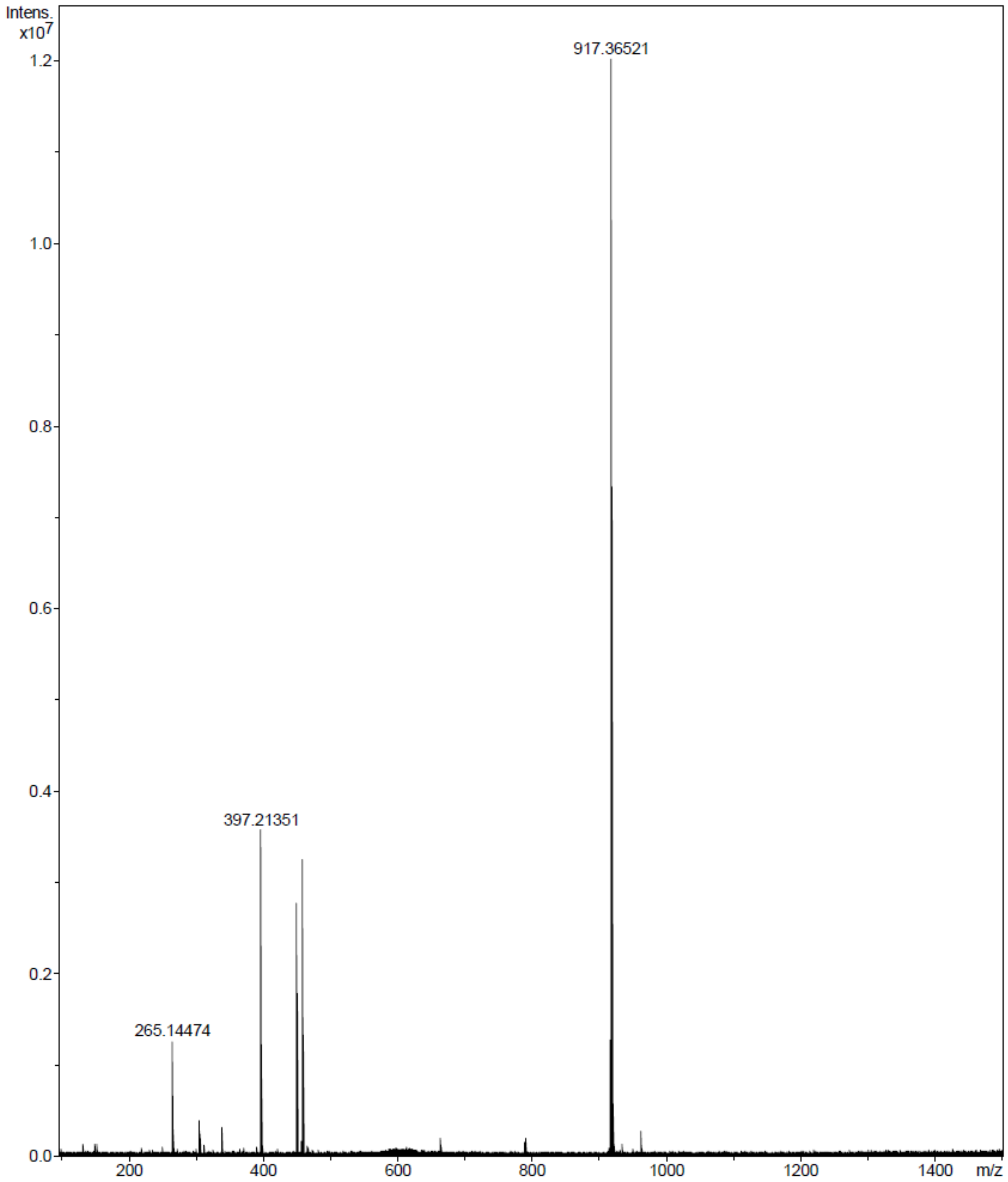


**Figure S15.** Electrospray ionization mass spectrum in positive-ion mode (HRMS-ESI+) of chlorin **7b**.

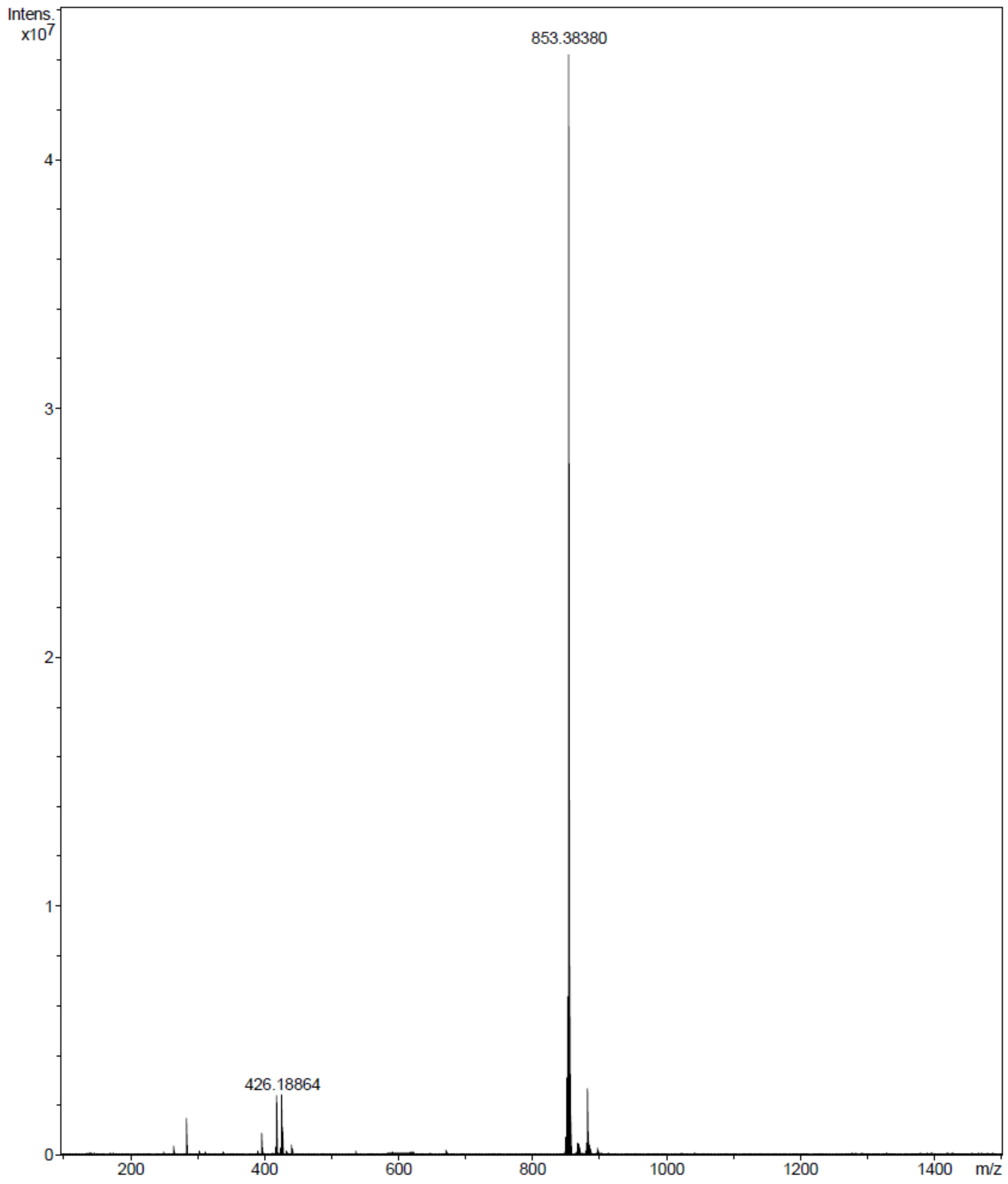


**Figure S16.** Electrospray ionization mass spectrum in positive-ion mode (HRMS-ESI+) of chlorin **7c**.

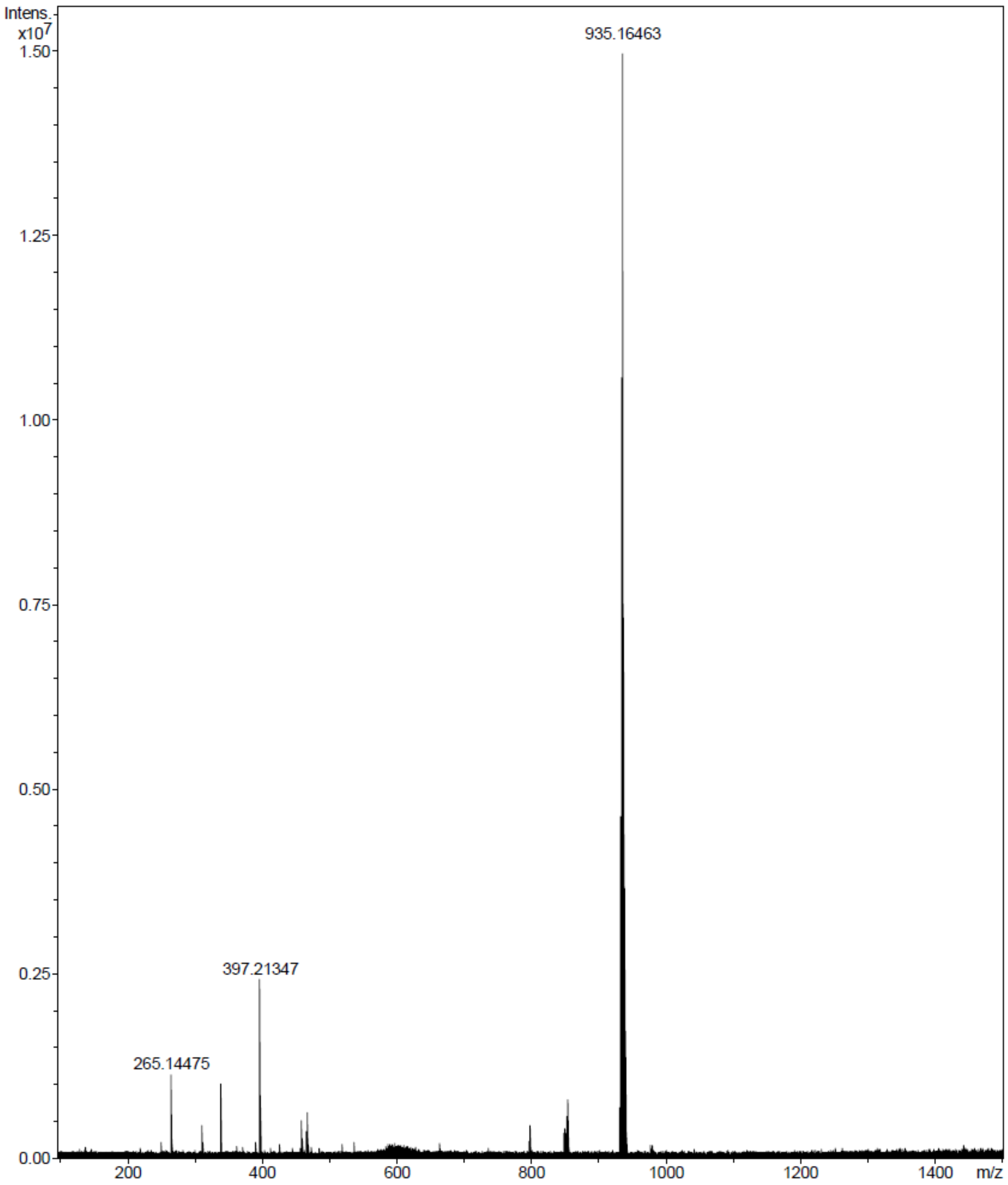


**Figure S17.** Electrospray ionization mass spectrum in positive-ion mode (HRMS-ESI+) of chlorin **7d**.

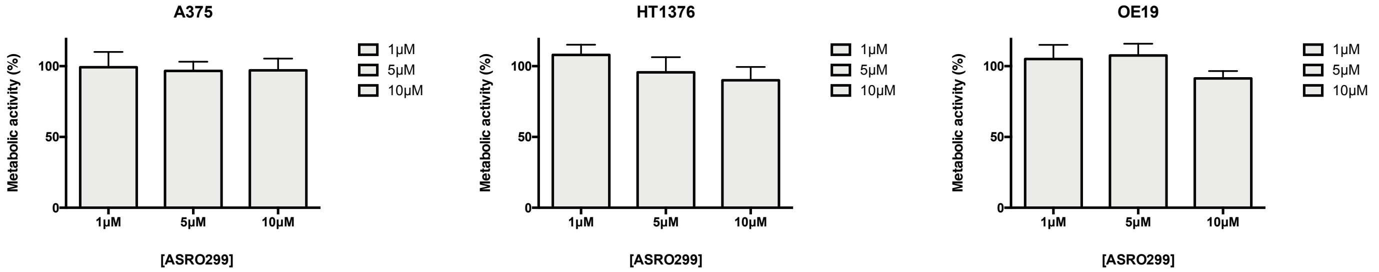


**Figure S18**. Cytotoxicity of chlorin **6b** in A375 skin malignant melanoma cells (left), HT1376 urinary bladder carcinoma cells (center), and OE19 esophageal adenocarcinoma cells (right). Cells were incubated with the chlorins and kept in the dark until the evaluation of metabolic activity. Results are presented as mean ± SD.

**Table S1.** Cytotoxicity of non-irradiated chlorin **6b** at different concentrations towards A375, HT1376 and OE19 tumor cells.

| [Chlorin 6b] (µM) | A375 | sd | HT1376 | sd | OE19 | sd |
|---|---|---|---|---|---|---|
| **0.05** | 100.6305 | 4.590397 | 108.556 | 4.608098 | 103.1781 | 5.9261 |
| **0.25** | 101.6604 | 9.919532 | 109.5435 | 5.72456 | 105.4278 | 8.213715 |
| **0.50** | 98.64598 | 8.640527 | 107.8084 | 7.305242 | 110.2971 | 6.09976 |
| **1** | 99.27281 | 10.79248 | 107.9424 | 7.187439 | 105.0067 | 10.08023 |
| **5** | 96.64089 | 6.461832 | 95.60141 | 10.76057 | 107.5996 | 8.289783 |
| **10** | 97.04092 | 8.310669 | 90.08365 | 9.37745 | 91.37507 | 5.207386 |

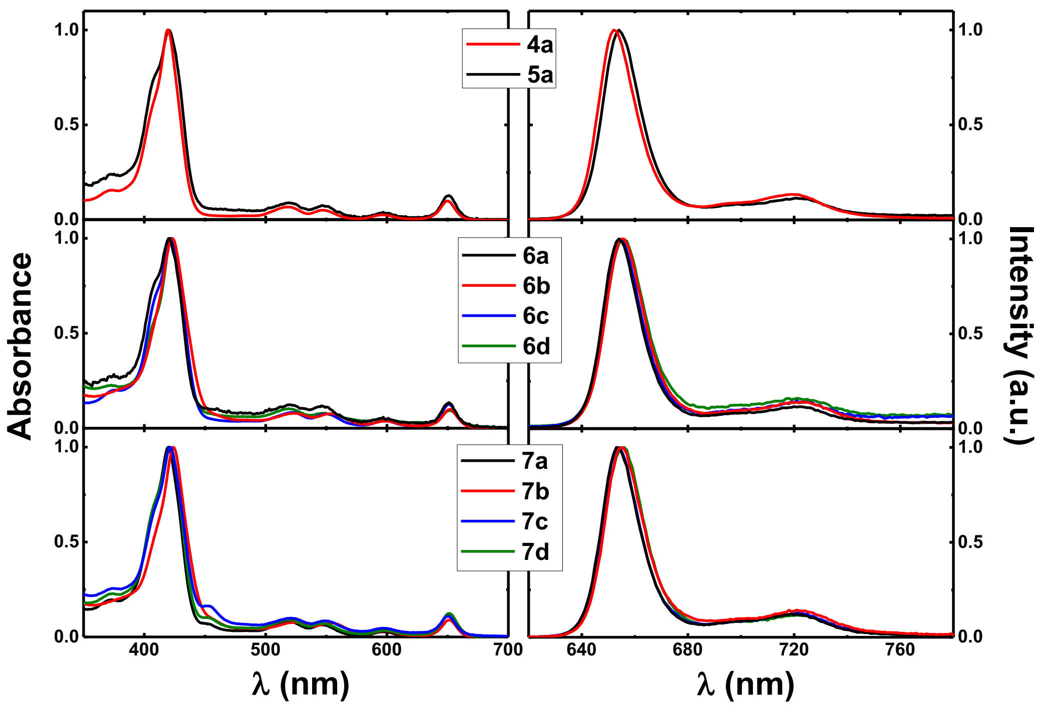


Figure S19. Normalized UV-Vis and fluorescence spectra of PS.
